# Transparent communication of evidence does not undermine public trust in evidence

**DOI:** 10.1093/pnasnexus/pgac280

**Published:** 2022-12-07

**Authors:** John R Kerr, Claudia R Schneider, Alexandra L J Freeman, Theresa Marteau, Sander van der Linden

**Affiliations:** Department of Psychology, School of Biological Sciences, University of Cambridge, Downing Street, CB2 3EB Cambridge, UK; Winton Centre for Risk and Evidence Communication, University of Cambridge, Wilberforce Road, CB3 0WA Cambridge, UK; Department of Psychology, School of Biological Sciences, University of Cambridge, Downing Street, CB2 3EB Cambridge, UK; Winton Centre for Risk and Evidence Communication, University of Cambridge, Wilberforce Road, CB3 0WA Cambridge, UK; Department of Psychology, School of Biological Sciences, University of Cambridge, Downing Street, CB2 3EB Cambridge, UK; Department of Public Health and Primary Care, University of Cambridge, Worts Causeway, CB1 8RN Cambridge, UK; Winton Centre for Risk and Evidence Communication, University of Cambridge, Wilberforce Road, CB3 0WA Cambridge, UK

**Keywords:** evidence communication, transparency, uncertainty, trust, trustworthiness, communication

## Abstract

Does clear and transparent communication of risks, benefits, and uncertainties increase or undermine public trust in scientific information that people use to guide their decision-making? We examined the impact of reframing messages written in traditional persuasive style to align instead with recent “evidence communication” principles, aiming to inform decision-making: communicating a balance of risks and benefits, disclosing uncertainties and evidence quality, and prebunking misperceptions. In two pre-registered experiments, UK participants read either a persuasive message or a balanced and informative message adhering to evidence communication recommendations about COVID-19 vaccines (Study 1) or nuclear power plants (Study 2). We find that balanced messages are either perceived as trustworthy as persuasive messages (Study 1), or more so (Study 2). However, we note a moderating role of prior beliefs such that balanced messages were consistently perceived as more trustworthy among those with negative or neutral prior beliefs about the message content. We furthermore note that participants who had read the persuasive message on nuclear power plants voiced significantly stronger support for nuclear power than those who had read the balanced message, despite rating the information as less trustworthy. There was no difference in vaccination intentions between groups reading the different vaccine messages.

Significance Statement A recently proposed set of five rules offers a guide for communicating evidence to facilitate decision-making, as opposed to trying to persuade the reader. However, some research suggests that the recommendations—such as acknowledging both risks and benefits or disclosing uncertainties—could have a negative impact on the perceived trustworthiness of the message and the messenger. Across two experiments, we compared texts written in traditional persuasive style against versions edited to be more balanced and transparent. The latter were not perceived as less trustworthy. In fact, for participants holding neutral or negative opinions about the message topic, such communications were perceived as more trustworthy. Taking a balanced, transparent approach to communicating evidence, therefore, does not undermine trustworthiness.

The notion that an individual or organization may seek to communicate information to an audience without the express intention of “getting a message across” and persuading them to act or think in a specific way is surprising, if not antithetical, to many professional communicators (1, 2). After all, authorities often seek to shift behavior through persuasive messages and “nudges”; governments usually aim to justify and garner public support for their policies; many subject-matter experts find themselves summarizing to a “take home message” to “get their point across.”

However, in many situations, there are strong practical, ethical, and legal reasons in which communicators, particularly experts, should explicitly not be aiming to change their audience’s behavior or opinion. Instead, their aim must be to provide balanced, clear, and understandable evidence to their audience, allowing them to weigh up the pros and cons and take into account relevant uncertainties in order to come to an informed decision of their own making rather than simply accept the opinion and interpretation of the communicator. One of the most obvious examples of this is in individual medical decision-making, where there is often a legal requirement that a patient be fully informed of potential treatments and their risks and benefits (3). However, although not always explicitly recognized, there are many other circumstances where this different, more transparent, kind of communication should be required. At the policy level, civil servants are expected to provide clear and balanced outlines of the costs and benefits of different policy options within government. This responsibility is often extended to external experts, as seen in the “honest broker” archetype of science advice (4). Clear communication of evidence for informed decision-making is also often the lynchpin of campaigns seeking to engage participatory decision-making with communities, e.g., on local environmental issues (5), and is crucial for effective referenda (6). Even when there is no statutory obligation to provide transparent and unbiased evidence, doing so is often defined as an honest signal of trustworthiness (7). While trusting is an act on the part of the audience, trustworthiness is a property of the communicator: Ideally, the degree of trust and trustworthiness of the two actors should be aligned but they are not necessarily so, as people can trust the untrustworthy and distrust the trustworthy. Honest cues of trustworthiness are, therefore, necessary to help people assess how to trust and weigh evidence in their decision-making (8).

The two aims—either to persuade the audience that the opinion of the communicator should be followed or to inform the audience about the evidence in as transparent a manner as possible to allow them to weigh it up for themselves—are very different and might often be in conflict with each other (9). A message designed to be maximally persuasive and entice readers toward one opinion will naturally minimize or even exclude evidence that does not support that opinion, or uncertainties that might undermine confidence in it. For communicators for whom “informing” is the aim, not “persuading,” the opinions and any decisions made by their audience after reading the information are of secondary importance compared with their understanding of, and trust in, the evidence presented.

To further empirical research on this issue, a recent article (10) outlined the contrasting aims of “informing” vs “persuading” and how messages designed to do the former might theoretically be constructed and evaluated, presenting “five rules” for trustworthy evidence communication, as summarized in Table 1.

**Table 1.** Overview of “five rules for evidence communication” from Blastland et al.

**Recommendation**	**Description**
Inform, not persuade	An overarching recommendation to communicate with the aim of informing the decision-maker’s choice, rather than pushing them towards a given option.
Offer balance, not false balance	Be clear about the benefits and costs or risks associated with decision options while acknowledging weight of evidence.
Disclose uncertainties	Clearly describe uncertainties around the evidence presented.
State evidence quality	Provide information about the quality of the evidence drawn upon.
Inoculate against misinformation	Identify and pre-empt circulating misinformation or misperceptions about the topic.

These recommendations were established based on the ethics of transparent and trustworthy communication, professional experience, as well as research across the fields of psychology, risk analysis, political science, and communication. The role of the cues of trustworthiness encapsulated within the recommendations is to help the audience know how much trust they can put in the facts contained within the communication, an important endpoint from an informed decision-making perspective and, on a longer timescale, for public trust in science and evidence more generally (11, 12). However, these cues of trustworthiness have not been empirically tested, as an ensemble, to see whether they achieve their aims. This study sets out to test them.

In order to do so, it is important to first understand how to measure what we would consider success in this context. The concepts of trust, credibility, expertise, and trustworthiness are defined in various, often overlapping, ways (13, 14). For example, some scholars frame credibility as consisting of two components: expertise (ability to make accurate assertions) and trustworthiness (willingness to communicate accurate assertions) (15). Others define trustworthiness as an overarching construct consisting of expertise, benevolence, and integrity (13). For the purposes of the current study, we conceptualize trustworthiness broadly, capturing expertise and competence, as well as benevolence and integrity.

While the communication of uncertainties, evidence quality, and pre-emptive corrections may be desirable from a purely theoretical perspective—as it makes the communication more trustworthy—it is unclear to what extent these elements actually act as cues of trustworthiness to a nonexpert, public audience. There is some empirical evidence on many of the cues in isolation, which suggests that they might be.

For example, a robust body of research in psychology and communication studies indicates that all things being equal, people perceive balanced, “two-sided” messages as more trustworthy than one-sided messages (16–20).

The majority of studies show that communication of statistical uncertainty, for instance, a numeric range around an estimate, does not reduce the trustworthiness of a message or its source (21, 22). However, some studies report that acknowledgment of large uncertainty intervals or unquantified uncertainties (e.g., simply stating that there is “some uncertainty”) can reduce trust in the presumed source of a message (21, 23, 24).

Beyond statistical uncertainty, communicators can also express uncertainty by disclosing the limitations or quality of the evidence available (25). Again, there are mixed results in the literature regarding the impact of this information on perceptions of trustworthiness. Empirical evidence suggests that messages which label claims as based on “low-quality” research are rated as less trustworthy than those which reference “high-quality” research or make no mention of quality (26, 27). Other studies have found that disclosure of evidence limitations by scientists in news or online reports can increase (28, 29) or have no effect on perceptions of credibility or trustworthiness (30, 31).

There are numerous studies examining the efficacy of fact-checks, prebunking, and debunking in countering misinformation and misperceptions, that is, they investigate the impact of such messages on belief in, or susceptibility to misinformation (32–34). But no studies have, to our knowledge, explicitly examined how the inclusion or exclusion of information aiming to correct or prebunk misinformation affects perceived trustworthiness of a message in and of itself.

In the current study, we aim to test whether messages that are produced according to principles of transparent evidence communication (10) are indeed perceived as more trustworthy by audiences than those in the more familiar, persuasive style (10). We do so empirically, by comparing realistic messages written in a persuasive style against the same messages edited to align with the transparent evidence communication principles (10) in two randomized, controlled trials.

We also set out to examine potential psychological reasons for any differences between the perceptions of the different messages. Previous research has shown that framing information as relating to a choice the reader must make, rather than as leading them to a particular outcome, is associated with less anger and negative cognitions, in line with psychological reactance theory (35, 36). Reactance, broadly defined, is a state of negative motivational arousal in response to threats to freedom. Where someone perceives a message as attempting to persuade them and limiting their freedom to choose, they may experience emotional (e.g., anger) and cognitive (e.g., mental counterarguing) responses to the message, which can ultimately push them towards the opposite attitudes or behaviors intended by the communicator, as means to restore a sense of freedom (37). In the current studies, we examine these emotional and cognitive facets of reactance alongside perceptions of trustworthiness.

To examine messaging effects in a realistic and real-world relevant context, we selected the topics of COVID-19 vaccines and nuclear power. Both are of high public salience and carry the possibility that some participants would hold strong views about these issues, which may affect their perceptions and allow us to investigate potential reactance effects caused by prior beliefs. People tend to ascribe more trustworthiness to information and sources that agree with their own views (38–41). It is possible that conflict with prior views could cause a net loss of trustworthiness when both pros and cons are presented. Thus, Blastland et al.’s (10) recommendations for trustworthy evidence communication could, theoretically, end up alienating those at either end of the attitude spectrum on a given issue. We investigate this possibility by examining how prior beliefs about message topics moderate the influence of message content on perceptions of trustworthiness and psychological reactance.

In the following studies, we take, as a starting point, communications produced by official sources that present a message written in a persuasive style. We then revised these in accordance with Blastland et al.’s (10) recommendations to produce a second version of each for comparison in two randomized, controlled studies. For simplicity, we refer to these two versions as the “Persuasive” and “Balanced” messages, respectively. However, we would stress that the Balanced messages not only provided a two-sided account of the pros and cons, but also included information about uncertainties, evidence quality, and potential misunderstandings, as per Blastland et al.’s recommendations. The process of editing to produce the balanced, evidence communication messages involved the authors collectively making judgments on what information to include to create a message that aligned with the five principles and contained reasonable examples of the suggested inclusions, whilst keeping the readability of the Persuasive and Balanced versions comparable. This approach does not allow us to isolate the effects of any one recommendation (e.g., disclosing uncertainty alone). Instead, our aim is to examine the net effect of the recommendations as a whole.

Participants in the experiments were randomized to read one of the message versions about either COVID-19 vaccines (Study 1) or a new nuclear power plant (Study 2) before responding to questions about the trustworthiness of the information and its (unnamed) source and completing measures of cognitive and affective reactance, that is, how they felt and thought about the message while reading it. In both studies, these outcomes—information trustworthiness, producer trustworthiness, cognitive reactance, and affective reactance—were pre-registered as our primary dependent variables. Results for a range of secondary outcomes are reported in the Supplementary Material.

## Study 1

This study was conducted in April 2021, prior to rollout of the COVID-19 vaccine to the general UK public under 50 years of age. In an online experiment, 2,928 UK adults who reported not yet having received a COVID-19 vaccine were recruited via the panel provider, Respondi, and randomly allocated to read one of three messages, which we label “Persuasive,” “Balanced,” and “Partial.” The Persuasive message was adapted from the UK’s National Health Service (NHS) website, encouraging vaccination against COVID-19, and describing the available vaccines simply as safe and effective. The Balanced message was a version of the text edited in line with Blastland et al.’s (10) recommendations for trustworthy evidence communication. This message was longer, but comparable to the Persuasive message in terms of readability (see the “Methods” section, full texts shown in Table S2). Briefly, this text: framed vaccination as a personal choice; included information on efficacy, side-effect frequency, uncertainties; and anticipated and corrected possible misperceptions. In the Partial condition, participants were presented with a minimally edited version of the Persuasive message including only one sentence for each of the five recommendations for trustworthy evidence communication. For reasons of brevity, and to simplify comparison with Study 2, which did not include a comparable condition, we report the results for the Partial condition in the Supplementary Material.

Before reading the messages, participants completed a measure of prior beliefs about COVID-19 vaccines captured on a 1 to 7 scale. The distribution was moderately negatively skewed (skew = −0.89) with most participants scoring above the midpoint of 4—i.e., generally agreeing that COVID-19 vaccines are safe and effective (*M* = 5.28, SD = 1.34).

Participants’ perceptions of trustworthiness were measured as the average of three items asking them the extent to which they found the information trustworthy, reliable, and accurate (21) (range 1 to 7). They were also asked to what extent they considered the producers of the information be trustworthy (range 1 to 7). There were no significant differences between the Persuasive and Balanced conditions in terms of how trustworthy participants rated the information (*M*_Persuasive_ = 5.28, SD = 1.50; *M*_Balanced_ = 5.39, SD = 1.35; *P = 0*.21, *d* = 0.08) or its producers (*M*_Persuasive_ = 5.16, SD = 1.53; *M*_Balanced_ = 5.28, SD = 1.36; *P = 0*.14, *d* = 0.09; pairwise comparisons are adjusted for multiple comparisons between conditions due to the presence of the third condition reported in the supplement, see the “Methods” section). To provide an indication of the strength of evidence for a null effect model given the data, we calculated Bayes factors for pairwise comparisons using the BayesFactor package (using a default Cauchy prior scaled by √2/2) (42). Results for the effect of condition on trustworthiness of the information, *BF*_10_ = 0.21, and producers, *BF*_10_ = 0.30, indicate “substantial” or “moderate” evidence in favor of the null model (43). There were no significant differences between the Persuasive and Balanced conditions in terms of affective reactance (i.e., anger; range 0 to 100; *M*_Persuasive_ = 17.50, SD = 24.43; *M*_Balanced_ = 15.47, SD = 21.51; *P = 0*.12, *d* = 0.09, *BF*_10_ = 0.32) or cognitive reactance (i.e., mental counter-arguing; range 1 to 7; *M*_Persuasive_ = 2.83, SD = 1.70; *M*_Balanced_ = 2.77, SD = 1.58; *P = 0*.70, *d* = 0.04, *BF*_10_ = 0.07).

**Fig. 1.**
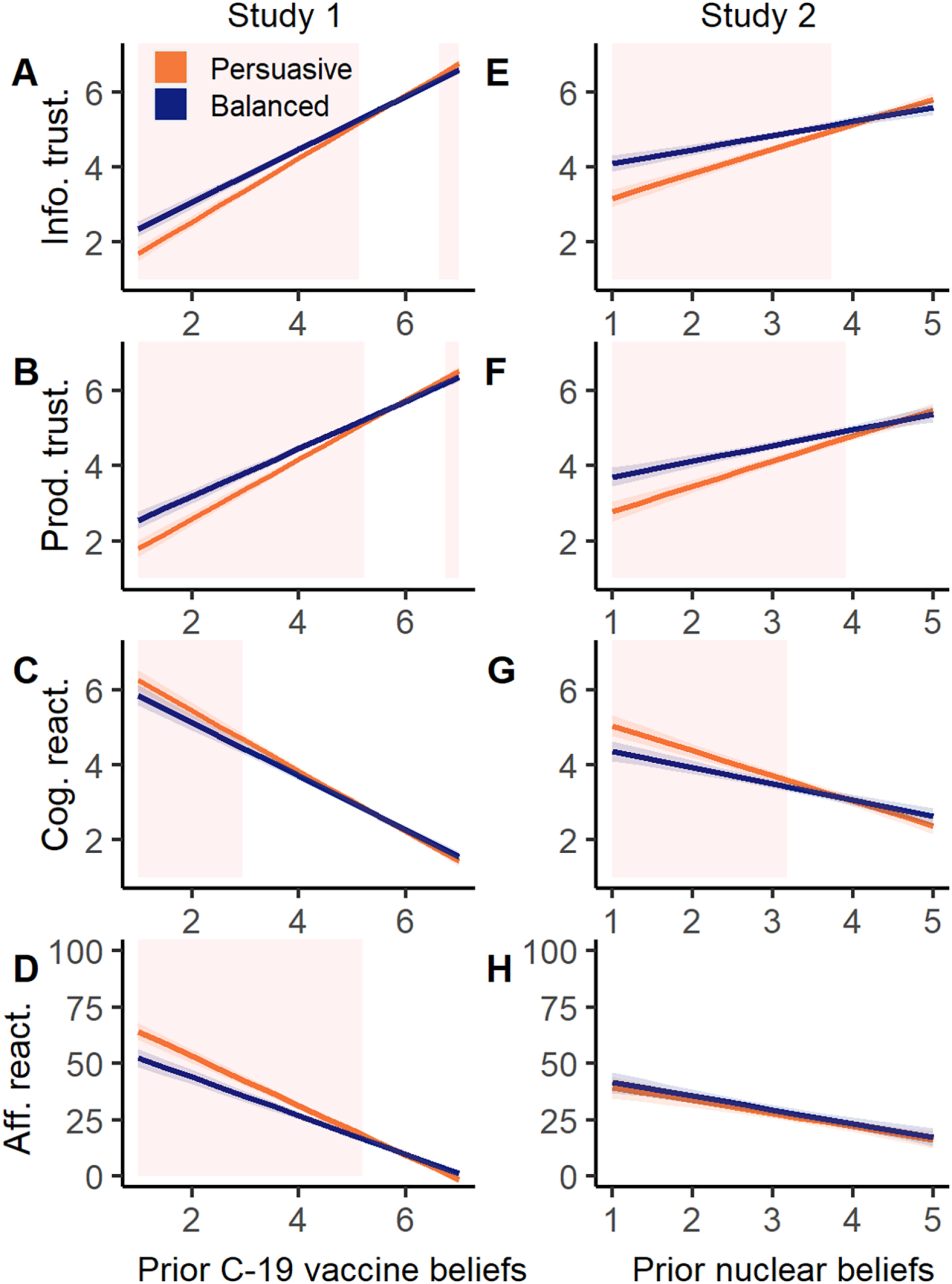
The moderating effect of prior beliefs on message effects in Study 1 (A to D) and Study 2 (E to H). Plots show predicted values (with 95% CI) of information trustworthiness (A, E), producer trustworthiness (B, F), cognitive (C, G), and affective reactance (D, H) in each condition across the spectrum of prior beliefs for study topic (higher values indicate more positive beliefs). The Balanced (vs Persuasive) condition has a greater effect on trustworthiness (positive effect) and, less consistently, reactance (negative effect) among participants with negative prior beliefs. Shaded regions indicate values of prior belief at which the effect of message condition is significant (*P* < 0.05), based on Johnson–Neyman intervals.

To examine the possibility that prior beliefs moderated the effect of message condition on perceived trustworthiness and reactance, we fitted a series of OLS regression models including an interaction term. We report significant interactions for all four outcomes, reported in Table 2 and Fig. 1A to D. Examination of the plots in Fig. 1 reveals that for all outcomes, there is little difference between message conditions when considering participants with positive prior beliefs regarding COVID-19 vaccines, that is, those believing that COVID-19 vaccines are safe and effective. However, when considering participants with negative prior beliefs about COVID-19 vaccines, we find that the Balanced message (vs Persuasive) is considered more trustworthy, and its producers are rated as more trustworthy. The Balanced message also elicited less cognitive and affective reactance among these participants. That is, they reported less mental counter-arguing and anger in response to the message.

**Table 2.** Study 1 interaction model results.

	**Information trustworthiness**	**Producer trustworthiness**	**Cognitive reactance**	**Affective reactance**
**Message (Persuasive vs Balanced)**	0.05	0.06	−0.01	−0.06
	[−0.01 to 0.11]	[−0.01 to 0.13]	[−0.08 to 0.06]	[−0.14 to 0.01]
Prior beliefs^a^	0.80***	0.74***	−0.66***	−0.64***
	[0.76 to 0.84]	[0.69 to 0.78]	[−0.71 to −0.61]	[−0.69 to −0.59]
Message*prior beliefs	−0.13***	−0.15***	0.07*	0.14***
	[−0.19 to −0.07]	[−0.21 to −0.08]	[0.00 to 0.14]	[0.07 to 0.21]
*N*	1,957	1,939	1,958	1,929
*R* ^2^ (adjusted)	0.545	0.450	0.393	0.336

Standardized regression coefficients shown with 95% CI in square brackets.

a Higher values indicate more positive beliefs.

*P < 0.05, ***P < 0.001.

To further investigate these interactions, we calculated Johnson–Neyman intervals to determine the values of the prior belief measures at which the effect of message condition is significant (*P* < 0.05; shaded regions in Fig. 1). The intervals for models predicting information trustworthiness [5.13, 6.63] and producer trustworthiness [5.23, 6.75] represent the range of values at which the effect of message is *not* significant. An interpretation of this is that at the most extreme positive end of the 1 to 7 prior beliefs scale—above 6.63 or 6.75 for information and producer trustworthiness, respectively—the Persuasive message is perceived as slightly more trustworthy than the Balanced message. In our sample, 14.2% of participants fell into this category (additional plots superimposing Johnson–Neyman intervals on the distribution of prior beliefs can be found in Fig. S1). However, as demonstrated in Fig. 1A and B, perceived trustworthiness on both measures remains very high across both groups for participants with very positive prior beliefs.

Participants also completed a measure of COVID-19 vaccination intention (44). Although not a primary outcome, we note no significant main effect of message condition on intentions, nor interaction with prior beliefs. Additionally, the Balanced message was rated by participants as taking slightly more effort to read (it was slightly longer), although there was no significant difference between conditions in terms of self-reported comprehension. Results for these additional measures included in the experiment are detailed in Tables S4 to S6.

A limitation of the design of this study is that we did not measure participants’ impressions of the texts in the context of trustworthy evidence communication recommendations (10). Did participants perceive the Balanced message as more balanced, open about uncertainty and so on, as intended? We also acknowledge a potential confound in the differing lengths of the Persuasive and Balanced texts. We sought to address these limitations in a second study focusing on a different topic.

## Study 2

**Fig. 2.**
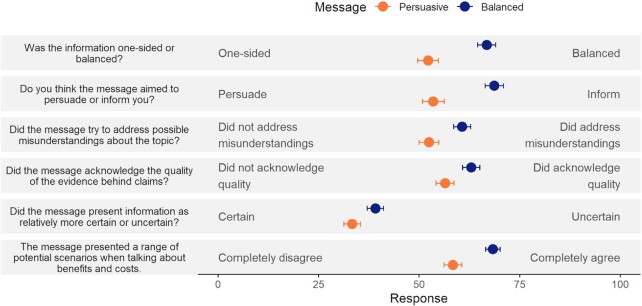
Cue detection results. Points show the mean responses (with 95% CI) across conditions for items capturing perceptions of the message meeting Blastland et al.’s recommendations, captured on a 0 to 100 sliding scale. Question text is shown at left and anchor labels for the 0 and 100 endpoints of each slider shown on plot. Error bars represent 95% CI.

In Study 2, we applied a slightly modified experimental design to the issue of building a new nuclear power plant in the United Kingdom. The nature of the decision was different; we presented a collective, policy decision—support for a new plant—as opposed to an individual decision—getting a vaccination. A total of 1,034 UK adults recruited via online panel provider Respondi completed the experiment. Participants first completed a measure of prior beliefs, capturing the extent to which they held a positive view of nuclear power in the United Kingdom (1 to 5 scale; *M* = 3.26, SD = 1.08; skew = −0.20). Participants were then randomly assigned to read one of two messages. The “Persuasive” message was based on extracts from recent UK Government policy documents, outlining arguments for building a nuclear power plant. The “Balanced” evidence communication message was edited as in Study 1 to present balanced information; detail uncertainties and evidence quality; and correct potential misperceptions, whilst remaining comparable in readability score. The texts were developed in consultation with subject-matter experts and piloted in a small study (see the “Methods” section). In Study 2, we included a set of items directly asking participants to rate the message they read according to the five principles for trustworthy evidence communication (10), for example, was the information one-sided or balanced? This gave us evidence of whether the cues that we intended to convey trustworthiness were indeed being noticed by the audience. Results are shown in Fig. 2. For all measures, the Balanced message was rated as more aligned with the recommendations (all *P* < 0.001, see Table S14). This, together with the similar lengths and readability scores, and participants’ similar ratings of the two messages on comprehensibility and ease of reading (see Supplementary Information), gave us some reassurance that the two messages were similar enough in other respects to provide an adequate test of the effects of the principles of trustworthy evidence communication (10).

Participants completed the same trustworthiness and reactance measures as in Study 1. Participants who read the Balanced message (vs Persuasive) rated the information as significantly more trustworthy (*M*_Persuasive_ = 4.70, SD = 1.33; *M*_Balanced_ = 4.92, SD = 1.19; two-sided Welch’s *t*-test: *P* < 0.01, *d* = 0.17, *BF*_10_ = 2.58) and its producers as significantly more trustworthy (*M*_Persuasive_ = 4.35, SD = 1.47; *M*_Balanced_ = 4.63, SD = 1.31; *P* < 0.01, *d* = 0.20, *BF*_10_ = 9.69). This differs from Study 1, where we report no main effect of the message condition on trustworthiness. In agreement with Study 1, we report no significant differences between message conditions in terms of affective reactance (*M*_Persuasive_ = 25.79, SD = 24.08; *M*_Balanced_ = 28.21, SD = 24.68; *P* = 0.11, *d* = 0.10, *BF*_10_ = 0.24) or cognitive reactance (*M*_Persuasive_ = 3.48, SD = 1.53; *M*_Balanced_ = 3.41, SD = 1.45; *P* = 0.41, *d* = 0.05, *BF*_10_ = 0.10).

As in Study 1, we fitted a series of OLS regression models examining the interaction between message condition and prior beliefs in predicting outcomes. Model results for these outcomes are reported in Table 3 and displayed graphically in Fig. 1. We report a similar pattern of effects to Study 1 when considering perceived trustworthiness of information and producers, and cognitive reactance. Among participants with negative prior beliefs about nuclear power, the Balanced message (vs Persuasive) and its producers were rated as more trustworthy, and the message elicited less cognitive reactance. Unlike Study 1, we did not find a significant interaction between message condition and prior beliefs in predicting affective reactance.

**Table 3.** Study 2 interaction model results.

	**Information trustworthiness**	**Producer trustworthiness**	**Cognitive reactance**	**Affective reactance**
**Message (Persuasive vs Balanced)**	0.22***	0.25***	−0.10	0.07
	[0.11 to 0.33]	[0.14 to 0.36]	[−0.21 to 0.01]	[−0.05 to 0.19]
Prior beliefs^a^	0.56***	0.52***	−0.48***	−0.26***
	[0.48 to 0.63]	[0.44 to 0.59]	[−0.56 to −0.40]	[−0.34 to −0.17]
Message*prior beliefs	−0.24***	−0.20***	0.17**	−0.01
	[−0.35 to −0.13]	[−0.31 to −0.09]	[0.06 to 0.28]	[−0.13 to 0.11]
*N*	1,030	1,034	1,033	1,034
*R* ^2^ (adjusted)	0.209	0.190	0.162	0.068

Standardized regression coefficients shown with 95% CI in square brackets.

a Higher values indicate more positive beliefs.

**P < 0.01, ***P < 0.001.

Participants also indicated their support for plans to build a new power plant in the United Kingdom. Although not a primary outcome, we note participants in the Persuasive (vs Balanced) condition expressed a significantly higher level of support for a new plant, and this effect was not moderated by prior beliefs. Results for additional measures included in the experiment are detailed in Tables S13 and S15.

## Discussion

Blastland et al.’s “five rules for evidence communication” (10) provide a broad set of recommendations for communicators seeking to inform an audience in a trustworthy fashion. We empirically measured the impact of following all of these recommendations together, on public perceptions of official messaging. Do people find messages that follow these rules and attempt to be purely informative more trustworthy than typical, persuasively written messages? Do they show less psychological reactance to messages that do not aim to be persuasive? And do they make different decisions after reading them?

One overarching conclusion we can draw from the two studies presented here is that framing information in a balanced way and acknowledging uncertainties do not substantively undermine the trustworthiness of a message or its source, compared to a more persuasive communication. This is an important finding in light of some concerns about more transparent communication of risk, benefits, and uncertainty (24, 45). But does such an approach *increase* perceptions of message trustworthiness? Considering only the main effects, the two studies give conflicting results. In Study 1, using messages about COVID-19 vaccines which were either one-sided and aiming to persuade or more balanced and seeking to inform, we find that on average people consider the information and source of either message to be equally trustworthy. In Study 2, communicating about nuclear power, we find that on average people consider the edited, balanced version of the message more trustworthy than the persuasive version. Thus, we find mixed evidence that messages adopting a balanced evidence communication approach are considered more trustworthy than messages aiming to persuade.

To some extent, these differing findings can be reconciled once we take into account how individuals’ pre-existing beliefs about the message topic influence their perceptions. In both experiments, we consistently find that the Balanced (vs Persuasive) message had little impact on trustworthiness measures for people with positive prior beliefs but among those with neutral or negative prior beliefs, the message and its producers were considered more trustworthy compared to the Persuasive message. Put another way, we show that such communications are perceived as more trustworthy among people with a sceptical view of the issue at hand. The differing distributions of prior beliefs in our studies can therefore explain the lack of main effects in Study 1. In Study 1, prior beliefs were skewed such that most of the sample had a positive view of COVID-19 vaccines and so showed little difference in trustworthiness ratings between the two messages. In Study 2, the distribution of pronuclear and antinuclear sentiment was much more even, giving a deeper pool of sceptical participants whose ratings of trustworthiness differed between the two messages. Although the effects in the current study are small, our results suggest that taking a balanced, evidence communication approach could reduce anger and mental counterarguing on the part of the audience when communicating information to sceptical groups (e.g., climate change deniers, antivaccination groups).

With regards to the cognitive and affective components of psychological reactance, i.e., theoretical precursors to “backfire” effects in persuasion, we find no overall differences in psychological reactance between people who read a persuading vs informing message across both studies. However, we again find that effects are moderated by prior beliefs. People with negative views about vaccines or nuclear power reported lower cognitive reactance in response to the Balanced message compared to the Persuasive message. We find a similar effect for affective reactance in Study 1 but not Study 2. The differing results here may be attributable to the emotional salience or personal importance of the content (46, 47). The individual decision to receive a vaccine is more immediate and psychologically proximal than a policy decision about nuclear infrastructure, and information about the vaccine may elicit stronger emotional reactions. We should also note that our measure of affective reactance required participants to rate their emotional response to the message using relatively strong terms such as “anger” and “bitterness.” In future research, measures including weaker terms such as “irritated” or “annoyed” could be used to capture milder negative affective responses with greater precision (37).

In both studies, message texts were framed around choices. Although not a key focus of the study, we do note that participants who read the Persuasive communication showed higher support for a nuclear power plant than those who read the Balanced version (Study 2). We did not find a difference in vaccine intentions in Study 1. Without knowing what participants’ decisions would have been prior to reading the communication, we cannot tell whether the persuasive message persuaded, or the informative message undermined support for a nuclear power plant—or both. We also stress that any effects of communications on such decision-related outcomes will be dependent on the risks, benefits, uncertainties, etc. included in or excluded from the message content. Nevertheless, the fact that there was a difference in average support between the experimental groups highlights the potential tension between informing and persuading (1): communicators seeking to persuade their audience may find that the transparent communication of risks, benefits, and uncertainties does not achieve this goal, or at least not as effectively as a more typical persuasive writing style.

Our findings align with a number of studies broadly showing that balanced, transparent communications are not perceived as less trustworthy than those that present a one-sided or more certain account (20, 22). However, direct comparisons to previous research are complicated not only by the multifaceted nature of the “five rules” for evidence communication (10), but also the ways in which these were implemented in our messages. For example, we disclosed uncertainty mainly in the form of numeric ranges, similar to a previous study, which also reports no effect of such uncertainty on trustworthiness (21), and as recommended by Blastland et al. (10). Other experiments have introduced uncertainty into communications through the presentation of conflicting or changing information (48, 49), in some cases reporting that this negatively affects the credibility of messages or their source (23, 50). Therefore, we cannot rule out the possibility that in some specific cases the application of Blastland et al.’s (10) recommendations in a broader sense could decrease perceptions of trustworthiness. Further, the current studies investigated the perceived trustworthiness of the communicators specifically, rather than overall trustworthiness of the medical or scientific community more broadly, as reported in some studies. (22) Future research could investigate such wider effects alongside the outcomes considered here.

One limitation of our study design was the inclusion of an attention check *after* the experimental manipulation in our survey experiments. Although condition assignment had no significant effect on failure rates in our studies, there is still a risk of bias when subsetting on post-treatment variables (51). For both studies reported here, repeating analyses with inattentive participants included produced comparable results (see the “Methods” section), indicating our results are not conditional on the removal of inattentive participants.

A further limitation of the current study is that we only tested the balanced, evidence communication approach against the messages written in persuasive style in one direction (i.e., provaccine and pronuclear messages). Future research could include two messages designed to persuade in opposing directions as well as a balanced “inform-only” message to further investigate whether symmetrical effects are seen, or whether there is a differential impact when choices are framed negatively, such as a message aiming to dissuade people from vaccination. Our Persuasive messages were drawn from existing communications, reflecting the typical information the UK public might see on the issues of vaccination and nuclear power. However, this approach means that these messages did not reflect the antithesis of the evidence communication approach captured in the Balanced message. Researchers seeking to create a more direct test of the recommendations could construct control messages with the aim to be as persuasive as possible—and piloted to ensure maximally persuasive messages are used—directing readers to a specific choice, while omitting any discussion of risks (or benefits, depending on the framing), evidence quality or uncertainty, and prebunking of misperceptions. This would provide a test with higher internal but lower external validity of the effect of the balanced, evidence communication approach on perceptions of trustworthiness.

In a similar vein, we treated transparency and persuasive intent as mutually exclusive in our experiments, since Blastland et al.’s (10) first recommendation was “aim to inform and not persuade.” However, a message could in theory be crafted that included all the information recommended by Blastland et al. (10) as well as an overt recommendation designed to guide the readers’ decision-making in one direction. The effect such messages have on their audiences could also be the subject of future studies.

We acknowledge that the manipulation used in the two studies we carried out was not tightly controlled, reflecting our aim to conduct an experiment examining the overall impact of combining Blastland et al.’s recommendations for trustworthy evidence communication, although we matched the messages for readability and, in Study 2, length. The fact that we find similar results with regards to the effects of balanced evidence communication in both studies points to a certain level of generalizability of the effect, which does not seem to depend on very tightly controlled subtle wording manipulations. As discussed in the Introduction, other research has investigated some components of the manipulations in isolation, but the current research is, to the best of our knowledge, the first to investigate the net effect of including these different kinds of information in a single message with the purpose of better informing decision-makers.

Future research should continue to investigate the extent to which these different message elements—including the persuasive/informative aims of the communicator—contribute to perceived trustworthiness. For example, experiments in which participants read a message or messages in which the separate elements investigated here, such as the presence or absence of uncertainty, are manipulated independently would be helpful. This would aid estimation of the unique effects of each on perceived trustworthiness and (with adequate power) interactions between them (52). We also encourage further research investigating the impact of transparent evidence communication on the perception of communications in other decision-relevant contexts to better understand the generalizability of the current results.

From a practical perspective, we must also note some challenges the evidence communication approach presents for communicators. Acknowledging both risks and benefits while disclosing uncertainties and evidence quality requires the addition of more information. This necessarily increases the length of communications and may increase the complexity for readers. Communicators should aim to incorporate such information in a clear and simple manner to minimize the effort required from the audience using formal readability indices to check this, alongside piloting information with the intended audience (53).

In conclusion, we find that balanced and transparent messages aiming to inform rather than to persuade are not perceived as less trustworthy. Indeed, it appears such message are perceived as more trustworthy and broadly elicit less psychological reactance among those with negative or neutral prior beliefs about the message content. These findings suggests that those wishing to communicate evidence in a way that is more trustworthy, and that is recognized as such by an audience with a broad range of prior views on a topic, should consider taking an “evidence communication” approach. This involves setting out to inform, with balanced, transparent information and discussion of uncertainties, rather than using traditional, persuasive writing styles. Conversely, those who wish to maximise their influence on their reader’s opinions and behavior may lose some of their audience’s trust. A proportion of the audience (particularly those with an opposing opinion on the topic beforehand) may see such a persuasive approach as less trustworthy.

## Methods

Both studies were approved by the Cambridge Psychology Research Ethics Committee (Study 1: PRE.2021.023; Study 2: PRE.2021.079) and preregistered with aspredicted.org (Study 1: https://aspredicted.org/rc956.pdf; Study 2: https://aspredicted.org/sv9mx.pdf). Two minor deviations from our pre-registered analyses are the use of Johnson–Neyman intervals (see Fig. 1) to further investigate the nature of reported interaction effects, and the use of Bayes factors to determine the strength of evidence for null treatment effects (requested during the peer-review process). Further details of study contexts and additional outcomes are provided in Supplementary Method.

### Study 1

#### Participants

Participants were recruited through the panel provider Respondi using quotas to match the sample to the UK population (aged 18 to 50) in terms of age and gender. Participants were paid approximately £1.60 for their participation. We set a target sample size of 2,454 participants for analysis, providing 95% power to detect a *d* = 0.2 effect size between conditions (*α* set at 0.016, incorporating a conservative Bonferroni correction due to a third condition, see below). This provides power to detect a “small” effect size (54) and is comparable to effect sizes reported in similar research investigating the effect of message content on perceived trustworthiness [see internal meta-analysis in van der Bles et al. (21)].

Participants who did not provide informed consent, reported being 50 years or older (because those older than 50 were mostly vaccinated at the time), or having received a COVID-19 vaccine, were screened from participating in the study. As pre-registered, participants failing an attention check (“please select agree”) embedded halfway through the survey were excluded from analysis [*N* = 301; failure rate did not differ between conditions, χ^2^(2) = 1.70, *P* = 0.42; results for analyses with all participants included were comparable to those reported in the main text, and the pattern of significant effects was unchanged, see Tables S7 to S9]. This resulted in a final sample size of *N* = 2,928 (*M*_Age_ = 33.7, SD = 9.55; 50.9% women; 82.3% reporting ethnicity as White; 56.8% reporting bachelor’s degree or higher; full demographic details are reported in Table S1).

Considering only the Persuasive and Balanced conditions reported in the main text, the final total sample size was *N* = 1,959 (*N*_Persuasive_ = 977; *N*_Balanced_ = 982).

#### Stimuli

Participants were asked to read one of three (two as the primary conditions reported in the main text, the third reported in the supplement) different messages about COVID-19. All messages were prefaced with a page explaining that the participant would be shown a short piece of information about COVID-19 vaccines and that they would be asked some questions about the content. No source information was provided.

The content of the messages is outlined in the following sections (full texts are provided in Table S2). All messages noted that: the vaccines were approved by regulators, the occurrence of serious side effects such as blood clots was extremely rare, and the vaccines do not contain animal or egg products.

**Persuasive:** This message was an abridged version of the information available to the public on the NHS website at the time of data collection (55). The message encouraged the reader to get vaccinated, describing the available COVID-19 vaccines simply as “safe and effective.” The message provided a bulleted list of potential minor side effects with no mention of frequency.

**Balanced:** This was based on the Persuasive version but edited through an iterative process among the authors, seeking to incorporate all five of the elements of balanced evidence communication outlined by Blastland et al. (10). Briefly, the message framed the information as there to help an individual choose whether or not to be vaccinated, rather than as reasons why they should choose vaccination. Evidence quality was communicated by stating that efficacy estimates and side effect frequencies were based on the results of clinical trials, and results from a “typical trial” were presented (56). The message outlined the sample size, absolute numbers of COVID-19 cases in the trial arms, efficacy calculations, and frequency of common side effects. The message also communicated uncertainty by noting the varying efficacy rates of vaccines (as a range of percentages) and unknown duration of immunity. Lastly, the message also aimed to pre-empt potential misunderstandings or misinformation by briefly explaining the accelerated regulatory review process and stating that the vaccines do not alter DNA or affect fertility (57).

Due to the addition of more information, the Balanced message text was longer than the Persuasive message text (289 and 542 words, respectively). However, the messages were comparable in readability [Flesch reading ease scores: Persuasive = 56.6, Balanced (excluding table) = 60.2].

A third “partial evidence communication” condition was included in the experiment. This version was only lightly edited according to the Blastland et al.’s (10) recommendations; only a single sentence was changed for each of the five points. This was not a “full” application of Blastland et al.’s (10) recommendations and, for brevity, we do not report results for this condition here (but see Supplementary Material). All pairwise comparisons account for multiple tests between the three conditions using Tukey’s post-hoc tests.

#### Measures

All items for the measures below are listed in Table S3. All multi-item scales demonstrated satisfactory reliability (Cronbach’s αs > 0.80, Table S4). Before reading the texts, participants completed a four-item measure of their prior beliefs about the safety and efficacy of COVID-19 vaccines (example item: *The currently available COVID-19 vaccines are safe*; 1 = *Strongly disagree*, 7 = *Strongly agree*). Information trustworthiness was captured as the average of participants’ ratings of the information as *trustworthy, accurate*, and *reliable* (1 = *Not at all*, 7 = *Very much*). Producer trustworthiness was measured with a single item asking participants the extent to which they thought the people who are responsible for producing this message were trustworthy (1 = *Not trustworthy at all*, 7 = *Very trustworthy*). Affective reactance was measured using the Aversion scale developed by Marcus et al. (58). This measure comprised the average of responses for the words *resentful, bitter, hateful*, and *angry* (sliding scale: 0 = *Not at all*, 100 = *Extremely*). Cognitive reactance was measured with Gardner and Leshner’s (59) three item scale (example:*Did you criticize the message you just saw while you were reading it?* 1 *= No, not at all*, 7 = *Yes, very much so*). Participants also reported their intentions to receive a COVID-19 vaccine via the Oxford Vaccine Hesitancy Scale (44) [example: *Would you take a COVID -19 vaccine (approved for use in the UK) if offered?* 1 = *Definitely*, 5 = *Definitely not*; scores reversed such that higher values indicate greater willingness].

### Study 2

#### Participants

Participants were recruited through the panel provider Respondi. We used a quota sampling approach to recruit a sample broadly representative of the UK population in terms of age and gender. Participants were paid approximately £1.50 for their participation.

We aimed for a sample size providing sufficient power to replicate the effect of the message by prior beliefs interaction on perceived trustworthiness reported in Study 1. Results of a series of simulations, using parameters from Study 1, indicated that a sample size of 1,300 would be sufficient to detect such an interaction term with 95% power (*α* = 0.05; see https://osf.io/4wcy3/). Due to budgetary constraints, we were not able to over-recruit to account for the removal of participants failing an attention check, therefore, the final sample size for analysis provided closer to 90% power to detect such an effect.

A total of 1,305 participants completed the survey. Of these, 271 (20.8%) failed an attention check embedded halfway through the survey. The failure rate did not differ between conditions, χ^2^(1) < 0.001, *P* = 0.997. Results for analyses with inattentive participants included were comparable to those reported in the main text, and the pattern of significant effects was unchanged, see Tables S16 and S17). This resulted in a final sample size of *N* = 1,034 (*M*_age_ = 47.2, SD = 16.1; 50.6% women; 89.9% reporting ethnicity as White; 43.5% reporting bachelor’s degree or higher; full demographic details reported in Table S10).

#### Stimuli

The content of the messages is outlined in the following sections (full texts are provided in Table S11).

**Persuasive:** A short, essentially pronuclear message was constructed based on excerpts from two UK policy documents: “Ten Point Plan for a Green Industrial Revolution” (60) and the 2020 “Energy white paper: Powering our net zero future” (61). The message first noted that the UK was considering building a new nuclear power plant, then explained that nuclear power was a reliable source of low-carbon energy and would help the United Kingdom achieve its net-zero 2050 goals. It outlined that construction could create 10,000 jobs and explained that the industry had committed to reducing the costs of construction by 30% by the end of the decade.

**Balanced:** As in Study 1, this message was based on the Persuasive version but heavily edited through an iterative process among the authors to incorporate all five of the elements outlined in Blastland et al. (10). Specifically, the adapted message presented a more balanced consideration of the choice by explicitly stating that there were potential benefits as well as potential downsides. In addition to the benefits noted in the Persuasive message, the Balanced message outlined negative aspects of nuclear power: high cost per energy unit compared to renewables, and challenges of safe operation and long-term waste storage. The message also acknowledged uncertainty through the inclusion of several hedge words (e.g., “could” rather than “will”), noting that employment impacts were not exact estimates, and providing numerical ranges (based on 95% CIs) for energy costs per energy unit (62). In terms of communicating evidence quality, the Balanced message noted that employment impacts were estimates based on previous projects and of low quality and that energy costs were based on government models. Lastly, the message addressed the misconception that nuclear power plants produce carbon emissions as part of their energy generation.

The Persuasive and Balanced messages were comparable in length and readability (322 and 313 words, respectively; Flesch reading ease score: Persuasive = 41.6, Balanced = 42.6). Study 2 messages and cue detection items, described in the next section, were first piloted with a small sample recruited via Respondi (*N* = 156, 51.3% male; *M*_age_ = 46.12, SD = 16.21), with participants randomly allocated to read one of the two messages. Although underpowered, results indicated that participants meaningfully differentiated the two messages on Blastland et al.’s criteria, using items with the same wording as those reported in Fig. 2. Analysis of open-ended comments did not raise any points of confusion regarding the content, and there were no significant differences between message groups in terms of reported effort required to read or comprehension of the texts.

We also sought feedback from several policy professionals familiar with nuclear power policy, asking them to check if the Persuasive message adequately reflected the source material from which it was taken. No concerns were raised.

#### Measures

All new items for the measures below are listed in Table S12. All multi-item scales demonstrated satisfactory reliability (Cronbach’s αs > 0.82, see Table S13).

Prior to reading, the experimental text participants completed Corner et al.’s (63) three-item measure of attitudes towards nuclear power in the United Kingdom (example item: *How favourable or unfavourable is your overall opinion or impression of nuclear power for producing electricity currently?* 1 = *Very favourable*, 5 = *Very unfavourable*; scores reversed such that higher values indicate a more positive disposition towards nuclear power).

Immediately following the presentation of the message, participants completed a set of items intended to check whether participants detected the changes that had been made to the Balanced message, relative to the Persuasive message. These items aimed to capture participants’ impressions of the message, framed in the wording of Blastland et al.’s five recommendations, with two separate items addressing uncertainty. These were presented as sliding scales (range 0–100) with endpoints labelled to reflect a spectrum presented by the item text. For example, participants were asked to rate the message on a scale from *one-sided* (0) to *balanced* (100). See Table S14 for all items and labels.

Participants then completed the following measures, all identical to Study 1: information trustworthiness, producer trustworthiness, affective reactance, cognitive reactance. Participants were also asked how much they support or oppose plans to build a new nuclear power plant in the United Kingdom (1 = *Strongly oppose*, 7 = *Strongly support*). Additional measures are detailed in Tables S3 and S12.

## Code Availability

The analysis R code developed for this study is available at https://osf.io/4wcy3/.

## Supplementary Material

pgac280_Supplemental_File

## Data Availability

The data used in this study are available at https://osf.io/4wcy3/.

## References

[1] VanDyke MS, Lee NM. 2020. Science public relations: the parallel, interwoven, and contrasting trajectories of public relations and science communication theory and practice. Public Relat Rev. 46:101953.

[2] Rossi J, Yudell M. 2012. The use of persuasion in public health communication: an ethical critique. Public Health Ethics. 5:192–205.

[3] Ward J et al. 2020. Shared decision making and consent post-Montgomery, UK Supreme Court judgement supporting best practice. Patient Educ Couns. 103:2609–2612. 10.1016/j.pec.2020.05.017 32451222

[4] Pielke RA . 2007. The honest broker: making sense of science in policy and politics. Cambridge: Cambridge University Press.

[5] Creighton JL . 2005. The public participation handbook. San Francisco (CA): Jossey-Bass.

[6] Renwick A, Palese M, Sargeant J. 2019. Information in referendum campaigns: how can it be improved?. Representation. 56:521–537.

[7] O'Neill O . 2018. Linking trust to trustworthiness. Int J Philos Stud. 26:293–300.

[8] O'Neill O 2020. Questioning trust. In: Simon J, editor. The Routledge handbook of trust and philosophy. New York: Routledge. p. 17–27.

[9] Oxman AD et al. 2022. Health communication in and out of public health emergencies: to persuade or to inform?. Health Res Policy Syst. 20:1–9. 35248064 10.1186/s12961-022-00828-z PMC8897761

[10] Blastland M, Freeman ALJ, van der Linden S, Marteau TM, Spiegelhalter D. 2020. Five rules for evidence communication. Nature. 587:362–364. 33208954 10.1038/d41586-020-03189-1

[11] Spatz ES, Krumholz HM, Moulton BW. 2016. The new era of informed consent: getting to a reasonable-patient standard through shared decision making. JAMA. 315:2063–2064. 27099970 10.1001/jama.2016.3070 PMC5459384

[12] Brick C et al. 2018. Winners and losers: communicating the potential impacts of policies. Palgrave Commun. 4:1–13. 10.1093/ntr/nty197 30312453

[13] Mayer RC, Davis JH, Schoorman FD. 1995. An integrative model of organizational trust. Acad Manage Rev. 20:734.

[14] Besley JC, Lee NM, Pressgrove G. 2020. Reassessing the variables used to measure public perceptions of scientists: Sci Commun. 43:3–32.

[15] Pornpitakpan C . 2004. The persuasiveness of source credibility: a critical review of five decades’ evidence. J Appl Soc Psychol. 34:243–281.

[16] Eisend M . 2006. Two-sided advertising: a meta-analysis. Int J Res Mark. 23:187–198.

[17] Mayweg-Paus E, Jucks R. 2017. Conflicting evidence or conflicting opinions? two-sided expert discussions contribute to experts’ trustworthiness. J Lang Soc Psychol. 37:203–223.

[18] Flanagin AJ, Winter S, Metzger MJ. 2018. Making sense of credibility in complex information environments: the role of message sidedness, information source, and thinking styles in credibility evaluation online. Inf Commun Soc. 23:1038–1056.

[19] Kamins MA, Marks LJ. 2013. Advertising puffery: the impact of using two-sided claims on product attitude and purchase intention. J Advert. 16:6–15.

[20] Petersen MB, Bor A, Jørgensen F, Lindholt MF. 2021. Transparent communication about negative features of COVID-19 vaccines decreases acceptance but increases trust. Proc Natl Acad Sci. 118:2024597118. 10.1073/pnas.2024597118 PMC8307373 34292869

[21] van der Bles AM, van der Linden S, Freeman ALJ, Spiegelhalter DJ. 2020. The effects of communicating uncertainty on public trust in facts and numbers. Proc Natl Acad Sci. 117:7672–7683. 32205438 10.1073/pnas.1913678117 PMC7149229

[22] Gustafson A, Rice RE. 2020. A review of the effects of uncertainty in public science communication. Public Understand Sci. 29:614–633. 10.1177/0963662520942122 32677865

[23] Han PK et al. 2018. Communication of scientific uncertainty about a novel pandemic health threat: ambiguity aversion and its mechanisms. J Health Commun. 23:435. 29648962 10.1080/10810730.2018.1461961 PMC6029253

[24] Kreps SE, Kriner DL. 2020. Model uncertainty, political contestation, and public trust in science: evidence from the COVID-19 pandemic. Sci Adv. 6:eabd4563. 32978142 10.1126/sciadv.abd4563 PMC7577608

[25] van der Bles AM et al. 2019. Communicating uncertainty about facts, numbers and science. R Soc Open Sci. 6:181870. 31218028 10.1098/rsos.181870 PMC6549952

[26] Schneider CR, Freeman ALJ, Spiegelhalter D, van der Linden S. 2021. The effects of quality of evidence communication on perception of public health information about COVID-19: two randomised controlled trials. PLoS One. 16:e0259048. 34788299 10.1371/journal.pone.0259048 PMC8598038

[27] Schneider CR, Freeman ALJ, Spiegelhalter D, van der Linden S. 2022. The effects of communicating scientific uncertainty on trust and decision making in a public health context. Judgm Decis Mak. 17:849–882.

[28] Jensen JD . 2008. Scientific uncertainty in news coverage of cancer research: effects of hedging on scientists’ and journalists’ credibility. Hum Commun Res. 34:347–369.

[29] Steijaert MJ, Schaap G, Riet JVT. 2021. Two-sided science: communicating scientific uncertainty increases trust in scientists and donation intention by decreasing attribution of communicator bias. Communications. 46:297–316.

[30] Ratcliff CL, Jensen JD, Christy K, Crossley K, Krakow M 2018. News coverage of cancer research : does disclosure of scientific uncertainty enhance credibility?. In: O'Hair HD, editor. Risk and health communication in an evolving media environment. New York (NY): Routledge. p. 156–175.

[31] Winter S, Krämer NC, Rösner L, Neubaum G. 2014. Don’t keep it (too) simple: how textual representations of scientific uncertainty affect laypersons’ attitudes. J Lang Soc Psychol. 34:251–272.

[32] Chan MS, Jones CR, Hall Jamieson K, Albarraci D. 2017. Debunking: a meta-analysis of the psychological efficacy of messages countering misinformation. Psychol Sci. 28:1531–1546. 28895452 10.1177/0956797617714579 PMC5673564

[33] Walter N, Cohen J, Holbert RL, Morag Y. 2019. Fact-checking: a meta-analysis of what works and for whom. Polit Commun. 37:350–375.

[34] Lewandowsky S, van der Linden S. 2021. Countering misinformation and fake news through inoculation and prebunking. Eur Rev Soc Psychol. 32:348–384.

[35] Rains SA . 2013. The nature of psychological reactance revisited: a meta-analytic review. Hum Commun Res. 39:47–73.

[36] Reynolds-Tylus T . 2019. Psychological reactance and persuasive health communication: a review of the literature. Front Commun (Lausanne). 4:56.

[37] Dillard JP, Shen L. 2005. On the nature of reactance and its role in persuasive health communication. Commun Monogr. 72:144–168.

[38] Nisbet EC, Cooper KE, Garrett RK. 2015. The partisan brain: how dissonant science messages lead conservatives and liberals to (dis)trust science. Ann Am Acad Pol Soc Sci. 658:36–66.

[39] Jacks JZ, Cameron KA. 2010. Strategies for resisting persuasion. Basic Appl Soc Psychol. 25:145–161.

[40] Kahan DM, Jenkins-Smith H, Braman D. 2011. Cultural cognition of scientific consensus. J Risk Res. 14:147–174.

[41] Metzger MJ, Hartsell EH, Flanagin AJ. 2015. Cognitive dissonance or credibility? A comparison of two theoretical explanations for selective exposure to partisan news. Commun Res. 47:3–28.

[42] Morey RD, Rouder JN. 2021. BayesFactor: computation of Bayes factors for common designs. R package version 0.9.12-4.3. [accessed 2022 Dec 12]. https://CRAN.R-project.org/package=BayesFactor.

[43] Steenbergen MR . 2019. What is in a (non-) significant finding? Moving beyond false dichotomies. Swiss Political Sci Rev. 25:300–311.

[44] Freeman D et al. 2021. COVID-19 vaccine hesitancy in the UK: the Oxford coronavirus explanations, attitudes, and narratives survey (Oceans) II. Psychol Med. 52:3127–3141. 10.1017/S0033291720005188 PMC7804077 33305716

[45] Fischhoff B . 2012. Communicating uncertainty: fulfilling the duty to inform. Issues Sci Technol. 28:63–70.

[46] Ball H, Wozniak TR. 2021. Why do some Americans resist COVID-19 prevention behavior? An analysis of issue importance, message fatigue, and reactance regarding COVID-19 messaging. Health Commun. 37:1812–1819. 33941005 10.1080/10410236.2021.1920717

[47] Quick BL, Scott AM, Ledbetter AM. 2011. A close examination of trait reactance and issue involvement as moderators of psychological reactance theory. J Health Commun. 16:660–679. 21391039 10.1080/10810730.2011.551989

[48] Lyons BA, Merola V, Reifler J. 2020. Shifting medical guidelines: compliance and spillover effects for revised antibiotic recommendations. Soc Sci Med. 255:112943. 32335462 10.1016/j.socscimed.2020.112943

[49] Simonovic N, Taber JM. 2022. Psychological impact of ambiguous health messages about COVID-19. J Behav Med. 45:159–171. 34811623 10.1007/s10865-021-00266-2 PMC8608560

[50] Shi W, Rothman AJ, Yzer MC, Nagler RH. 2022. Effects of exposure to conflicting information about mammography on cancer information overload, perceived scientists’ credibility, and perceived journalists’ credibility. Health Commun. 1–10.. [accessed 2022 Dec 12]. 10.1080/10410236.2022.2077163. 35607276 PMC9681936

[51] Montgomery JM, Nyhan B, Torres M. 2018. How conditioning on posttreatment variables can ruin your experiment and what to do about it. Am J Pol Sci. 62:760–775.

[52] Priem RL, Weibel AA. 2015. Measuring the decision to trust using metric conjoint analysis. In: Lyon F, Möllering G, Saunders M, editors. Handbook of research methods on trust. 2nd ed. Cheltenham: Elgar. p. 265–278.

[53] Freeling BS, Doubleday ZA, Dry MJ, Semmler C, Connell SD. 2021. Better writing in scientific publications builds reader confidence and understanding. Front Psychol. 12:3484. 10.3389/fpsyg.2021.714321 PMC8430246 34512473

[54] Schäfer T, Schwarz MA. 2019. The meaningfulness of effect sizes in psychological research: differences between sub-disciplines and the impact of potential biases. Front Psychol. 10:813. 31031679 10.3389/fpsyg.2019.00813 PMC6470248

[55] NHS . 2021. Coronavirus (COVID-19) vaccine (archived). [accessed 2022 Dec 12]. https://web.archive.org/web/20210408070250/https://www.nhs.uk/conditions/coronavirus-covid-19/coronavirus-vaccination/coronavirus-vaccine/.

[56] FDA . 2020. FDA briefing document: Moderna COVID-19 vaccine. [accessed 2022 Dec 12]. https://www.fda.gov/media/144434/download.

[57] An L et al. 2021. Online search behavior related to COVID-19 vaccines: infodemiology study. JMIR Infodemiology. 1:e32127. 34841200 10.2196/32127 PMC8601025

[58] Marcus GE, Neuman WR, MacKuen MB. 2017. Measuring emotional response: comparing alternative approaches to measurement. Political Sci Res Methods. 5:733–754.

[59] Gardner L, Leshner G. 2015. The role of narrative and other-referencing in attenuating psychological reactance to diabetes self-care messages. Health Commun. 31:738–751. 26528578 10.1080/10410236.2014.993498

[60] HM Government . 2020. The ten point plan for a green industrial revolution. [accessed 2022 Dec 12]. https://www.gov.uk/government/publications/the-ten-point-plan-for-a-green-industrial-revolution.

[61] Department for Business Energy & Industrial Strategy . 2020. Energy white paper: powering our net zero future. Energy Department, 44. [accessed 2022 Dec 12]. https://www.gov.uk/government/publications/energy-white-paper-powering-our-net-zero-future.

[62] Evans S . 2020. Wind and solar are 30–50% cheaper than thought, admits UK government. Carbon Brief. [accessed 2022 Dec 12]. https://www.carbonbrief.org/wind-and-solar-are-30-50-cheaper-than-thought-admits-uk-government/.

[63] Corner A et al. 2011. Nuclear power, climate change and energy security: exploring British public attitudes. Energy Policy. 39:4823–4833.

