# Correction to: Transparent communication of evidence does not undermine public trust in evidence

**DOI:** 10.1093/pnasnexus/pgad476

**Published:** 2024-01-30

This is a correction to: John R Kerr, Claudia R Schneider, Alexandra L J Freeman, Theresa Marteau, Sander van der Linden, Transparent communication of evidence does not undermine public trust in evidence, *PNAS Nexus*, Volume 1, Issue 5, November 2022, pgac280, https://doi.org/10.1093/pnasnexus/pgac280

During a retroactive audit conducted by *PNAS Nexus*, it was discovered that this paper was missing a statement acknowledging compliance with the *PNAS Nexus* Human and Animal Participants and Clinical Trials policy:

All participants in both studies provided informed consent, as per the protocols approved by the Cambridge Psychology Research Ethics Committee.

This error has been corrected in the original article.

