# Supplementary material for: Transparent communication of evidence does not undermine public trust in evidence
Source: PNAS Nexus. 2022 Dec 7;1(5):pgac280. doi: 10.1093/pnasnexus/pgac280 (PMC9802351; doi:10.1093/pnasnexus/pgac280)
Supplement: pgac280_Supplemental_File [file pgac280_supplemental_file.docx]

**Transparent communication of evidence does not undermine public trust in evidence**

**SUPPLEMENTARY MATERIALS**

Contents

[Supplementary Methods 2](#_Toc117684403)

[Study 1 2](#_Toc117684404)

[Context 2](#_Toc117684405)

[Stimuli 2](#_Toc117684406)

[Measures 2](#_Toc117684407)

[Study 2 3](#_Toc117684408)

[Context 3](#_Toc117684409)

[Stimuli 4](#_Toc117684410)

[Measures 4](#_Toc117684411)

[References 5](#_Toc117684412)

[Supplementary tables 7](#_Toc117684413)

[**Supplementary table 1.** Study 1 demographics 7](#_Toc117684414)

[**Supplementary table 2.** Full texts of stimuli in Study 1 8](#_Toc117684415)

[**Supplementary table 3.** Study 1 questionnaire wording 12](#_Toc117684416)

[**Supplementary table 4.** Scale reliabilities, means and standard deviations of Study 1 outcomes, across conditions 17](#_Toc117684417)

[**Supplementary table 5.** Study 1 results of one-way ANOVA tests and pairwise comparisons between conditions 18](#_Toc117684418)

[**Supplementary table 6.** Study 1 full regression results for models examining interaction between message condition and prior beliefs 20](#_Toc117684419)

[**Supplementary table 7.** Study 1 group means (including inattentive participants). 21](#_Toc117684420)

[**Supplementary table 8.** Study 1 main effects (including inattentive participants). 21](#_Toc117684421)

[**Supplementary table 9.** Study 1 interaction effects (including inattentive participants). 22](#_Toc117684422)

[**Supplementary table 10.** Study 2 demographics 23](#_Toc117684423)

[**Supplementary table 11.** Full texts of stimuli in Study 2 24](#_Toc117684424)

[**Supplementary table 12.** Study 2 additional questionnaire items wording 26](#_Toc117684425)

[**Supplementary table 13.** Scale reliabilities, means and standard deviations of Study 2 outcomes, across conditions, and t-test results. 28](#_Toc117684426)

[**Supplementary table 14.** Study 2 cue detection slider item details, condition means and SD, and t-test results. 29](#_Toc117684427)

[**Supplementary table 15.** Study 2 full regression results for models examining interaction between message condition and prior beliefs in predicting outcomes. 30](#_Toc117684428)

[**Supplementary table 16.** Study 2 group means and main effects (including inattentive participants) 31](#_Toc117684429)

[**Supplementary table 17.** Study 2 interaction effects (including inattentive participants) 31](#_Toc117684430)

[Supplementary figures 32](#_Toc117684431)

[**Supplementary Figure 1.** Study 1 interaction plots with distribution of prior belief scores. 32](#_Toc117684432)

# Supplementary Methods

## Study 1

### Context

This study was undertaken in April 2021, during the initial COVID-19 vaccine rollout in the UK. At the time of data collection, UK adults over 50 and the clinically vulnerable were eligible to receive Moderna or AstraZeneca vaccines, and a majority of people in these groups had already received a vaccination (NHS, 2021a., 2021b). As the experimental messages related to the decision to receive a vaccine or not, we aimed to survey individuals who had not yet received a vaccine but were likely to be asked in the near future. We therefore limited our sample to those under 50 and excluded participants who reported having already received a COVID-19 vaccination. Three COVID-19 vaccines were approved for use in the UK at the time of the survey: AstraZeneca, Pfizer-BioNTech BNT162b2 and Moderna mRNA-1273. We note that at the time of data collection there had been media coverage of a possible link between the AstraZeneca COVID-19 vaccine and rare instances of a particular type of blood clot (e.g., Cuffe, 2021). This potential link was acknowledged in both versions of the message provided to participants, as it was present in the NHS source material described below.

### Stimuli

As noted in the Methods section of the main text, this experiment included an additional, pre-registered condition in which participants read a version of the Persuasive message with only minor changes to the text attempting to address the Blastland et al (2020) guidelines (‘Partial’ condition). The message text is detailed in Supplementary table 2, and results for this condition are reported alongside the main results in Supplementary tables 4, 5, and 6.

### Measures

For completeness we report here additional items and scales included in the survey experiment. All items for the measures below are listed in Supplementary table 3. Participants completed Dillard and Shen’s (2005) four item scale capturing perceived threats to freedom of choice (example: *The message tried to manipulate me*; 1 = *Strongly disagree*, 7 = *Strongly agree*). Perceived quality of evidence was measured with a single item: *How high or low do you think the quality of the evidence underlying the information you just read is?* (1 = *Low quality of eviden*ce, 7 = *High quality of evidence*). Participants rated the message in terms of: engagement (three items; example: *In your opinion, would other people want to read this?*; 1 = *Not at all*, 7 = *Very much*), understanding (1 = *Didn’t understand it all*, 7 = *Understood it completely*), and effort required to read (1 = *None*, 7 = *A lot*).

Participants also reported their intentions to receive a COVID-19 vaccine. This was measured using Freeman et al.’s (2021) seven item Oxford Vaccine Hesitancy Scale (example: *Would you take a COVID-19 vaccine (approved for use in the UK) if offered?* 1 = *Definitely*, 5 = *Definitely not*; scores reversed such that higher values indicate greater willingness). Vaccine intentions were also measured with a simple binary item. Participants provided a yes or no response to the question: *If you were offered a COVID-19 vaccine, would you get vaccinated yourself?*

Finally, participants completed the full 16 item Decisional Conflict Scale (O’Connor, 1995). Items were prefaced with the preamble*: If you were given the option of receiving a COVID-19 vaccine or not today how would you feel about the decision?* Due to the wide-ranging nature of the scale and for direct comparison with Study 2, we report here the average of four items deemed most relevant to a participant’s vaccination decision considering the information provided (*I feel sure about what to choose*; *This decision is easy for me to make*; *I feel I have made an informed choice*; *I am satisfied with my decision*; 1 = *Strongly disagree*, 5 = *Strongly agree*). For completeness, we also report results using the full 16-item scale, which are comparable.

## Study 2

### Context

The content of the messages in this study focused on the UK Government’s decision to invest in the building of a new nuclear power plant. This was a topic of national interest expected to elicit a diversity of views among the public, not clearly split along political party or environmental lines. The government had signalled its intention to invest in a further power plant in previous policy documents (Department for Business Energy & Industrial Strategy, 2020; HM Government, 2020), framing nuclear power as part of a strategy to reach net zero carbon emissions by 2050. The COP26 climate conference was taking place at the time of data collection and the prospect of a new power plant had received national media attention (Harrabin, 2021).

### Stimuli

The messages used in this study are detailed in Supplementary table 8.

### Measures

In addition to the measures reported in the main text, participants completed the following measures, all identical to Study 1: engagement, perceived quality of evidence, difficulty, and effort required to read.

Participants were also asked how much they support or oppose plans to build a new nuclear power plant in the UK (1 = *Strongly oppose*, 7 = *Strongly support*). Following this, participants completed the four items from the Decisional Conflict Scale from Study 1, with specific reference to their decision to oppose or support a new plant.

## References

Blastland, M., Freeman, A. L., van der Linden, S., Marteau, T. M., & Spiegelhalter, D. (2020). Five rules for evidence communication. *Nature,* 587, 362-364. https://doi.org/10.1038/d41586-020-03189-1

Cuffe, R. (2021). *AstraZeneca vaccine: How do you weigh up the risks and benefits? - BBC News*. BBC News. https://www.bbc.co.uk/news/explainers-56665396

Department for Business Energy & Industrial Strategy. (2020). Energy white paper: Powering our net zero future. In APS Group on behalf of the Controller of Her Majesty’s Stationery Office (Ed.), *Energy Department* (Vol. 44, Issue December). https://www.gov.uk/government/publications/energy-white-paper-powering-our-net-zero-future

Dillard, J. P., & Shen, L. (2005). On the Nature of Reactance and its Role in Persuasive Health Communication. *Communication Monographs* , *72*(2), 144–168. https://doi.org/10.1080/03637750500111815

Freeman, D., Loe, B. S., Chadwick, A., Vaccari, C., Waite, F., Rosebrock, L., Jenner, L., Petit, A., Lewandowsky, S., Vanderslott, S., Innocenti, S., Larkin, M., Giubilini, A., Yu, L. M., McShane, H., Pollard, A. J., & Lambe, S. (2021). COVID-19 vaccine hesitancy in the UK: The Oxford coronavirus explanations, attitudes, and narratives survey (Oceans) II. *Psychological Medicine*, 1–15. https://doi.org/10.1017/S0033291720005188

Harrabin, R. (2021, October 19). *Net zero announcement: UK sets out plans to cut greenhouse gas emissions*. BBC News. https://www.bbc.co.uk/news/science-environment-58899006

HM Government. (2020). *The ten point plan for a green industrial revolution*. https://www.gov.uk/government/publications/the-ten-point-plan-for-a-green-industrial-revolution

NHS. (2021). *COVID-19 Vaccination Statistics Week ending Sunday 25th April 2021* . https://www.england.nhs.uk/statistics/wp-content/uploads/sites/2/2021/04/COVID-19-weekly-announced-vaccinations-29-April-2021.pdf

NHS. (2021). *Who can get the coronavirus (COVID-19) vaccine (archived)*. Retrieved January 28, 2022, from https://web.archive.org/web/20210409070935/https://www.nhs.uk/conditions/coronavirus-covid-19/coronavirus-vaccination/who-can-get-the-vaccine/

O’Connor, A. M. (1995). Validation of a Decisional Conflict Scale. *Medical Decision Making*, *15*(1), 25–30. https://doi.org/10.1177/0272989X9501500105

# Supplementary tables

### **Supplementary table 1.** Study 1 demographics

| Category | Response | n | % |
| --- | --- | --- | --- |
| Gender | Woman | 1416 | 48.4 |
|  | Man | 1491 | 50.9 |
|  | Other | 13 | 0.4 |
|  | I prefer not to answer | 8 | 0.3 |
| Age bracket^a^ | 18-24 | 568 | 19.4 |
|  | 25-34 | 999 | 34.1 |
|  | 35-44 | 824 | 28.1 |
|  | 45-50 | 537 | 18.3 |
| Ethnicity | Arab | 16 | 0.5 |
|  | Asian | 288 | 9.8 |
|  | Black | 85 | 2.9 |
|  | Mixed | 94 | 3.2 |
|  | Other | 34 | 1.2 |
|  | White | 2411 | 82.3 |
| Education | No formal education above age 16 | 163 | 5.6 |
|  | Professional or technical qualifications above age 16 | 275 | 9.4 |
|  | School education up to age 18 | 828 | 28.3 |
|  | Degree (Bachelors) or equivalent | 1130 | 38.6 |
|  | Degree (Masters) or equivalent | 469 | 16.0 |
|  | Doctorate | 63 | 2.2 |

^a^Participants also reported their age in years (*M* =33.7, *SD* = 9.55)

### **Supplementary table 2.** Full texts of stimuli in Study 1

| Condition | Message text |
| --- | --- |
| Persuasive | **COVID-19 (coronavirus) vaccine**  The COVID-19 vaccine is safe and effective. It gives you the best protection against COVID-19.  **How safe is the COVID-19 vaccine?**  The vaccines approved for use in the UK have met strict standards of safety, quality and effectiveness set out by the independent Medicines and Healthcare products Regulatory Agency (MHRA).  Any COVID-19 vaccine that is approved must go through all the clinical trials and safety checks all other licensed medicines go through. The MHRA follows international standards of safety.  Other vaccines are being developed. They will only be available on the NHS once they have been thoroughly tested to make sure they are safe and effective.  So far, millions of people have been given a COVID-19 vaccine and reports of serious side effects, such as allergic reactions or blood clots, have been very rare. No long-term health complications have been reported.  **How effective is the COVID-19 vaccine?**  The first dose of the COVID-19 vaccine should give you good protection from COVID-19. But you need to have the 2 doses of the vaccine to give you longer lasting protection.  There is a chance you might still get or spread COVID-19 even if you have the vaccine. This means it is important to:   - continue to follow social distancing guidance, - if you can, wear something that covers your nose and mouth in places where it's hard to stay away from other people.   **COVID-19 vaccine side effects**  Most side effects of the COVID-19 vaccine are mild and should not last longer than a week, such as: a sore arm where the needle went in,   - feeling tired, - a headache, - feeling achy, - feeling or being sick   **COVID-19 vaccine ingredients**  The approved COVID-19 vaccines do not contain any animal products or egg. |
| Balanced | **COVID-19 (coronavirus) vaccination: making your decision**  The COVID-19 vaccine is now being offered in the UK. This information is designed to help you make an informed decision about vaccination.  All medical treatments have potential benefits and potential side effects which should be considered.  **Rapid approvals process**  Any COVID-19 vaccine that is approved must go through all the clinical trials and safety checks all other licensed medicines go through.  However, the medicines regulators have provided ‘rolling review’. This means that they have been able to assess the data at it has come in, to speed up the authorisation application assessment.  **Potential benefits of the COVID-19 vaccine**  The vaccines are designed to protect you against becoming ill with COVID-19.  The vaccines available in the UK have all been through similar testing. In a typical clinical trial, one of the vaccines has been tested in over 10,500 volunteers aged 18-64 and over 3,500 over 65s, including many ethnicities and people with underlying health conditions. These volunteers were compared with the same number of people who got a dummy (placebo) injection.  During the course of the trial, people in both groups tested positive for Covid-19.   - 7 people who had the vaccine got COVID-19 - 156 people who had the dummy injection got COVID-19.   This means that there were 94% fewer cases of COVID-19 in those who have been vaccinated (86% in the over-65s). Researchers think that the vaccine will prevent somewhere between 89% to 97% of cases overall.  The other vaccines available were tested in very similar ways and with very similar outcomes.  It is not currently known how long protection lasts. The people vaccinated in the clinical trial will continue to be followed for 2 years.  There is a chance you might still get sick or spread COVID-19 even if you have the vaccine.  This means it is important to:   - continue to follow social distancing guidance, - if you can, wear something that covers your nose and mouth in places where it's hard to stay away from other people.   **COVID-19 vaccine side effects**  Most side effects of the COVID-19 vaccine are mild and should not last longer than a week. In clinical trials, certain side effects were more common for people who received the vaccine compared to those who received a dummy (placebo) injection:   \| Side effect \| Percent of people reporting side effect in clinical trial \| \| \| --- \| --- \| --- \| \|  \| Received a vaccine \| Received a dummy injection \| \| a sore arm where the needle went in \| 90% \| 19% \| \| feeling tired \| 68% \| 36% \| \| a headache \| 63% \| 36% \| \| feeling achy \| 60% \| 20% \| \| feeling or being sick \| 21% \| 7% \|   So far, 21 million people in the UK received at least the first dose of a COVID-19 vaccine and reports of serious possible side effects, such as allergic reactions or blood clots, have been very rare (between 3 and 5 reports per 10,000 vaccinations).  **How vaccines work**  Our natural immune system helps us fight infection from harmful bacteria or viruses by creating antibodies. Vaccines work by triggering our bodies to produce antibodies without the actual dangerous bacteria or virus itself being there.  There are lots of myths about the vaccine: you cannot get COVID-19 from the vaccine, it does not affect your DNA, nor your fertility. The vaccines also do not contain any animal products or egg. |
| Partial (results not reported in main text) | **COVID-19 (coronavirus) vaccine**  The COVID-19 vaccine is now being offered in the UK. This information is designed to help you make an informed decision about vaccination.  All medical treatments have potential benefits and potential side effects which should be considered.  **How safe is the COVID-19 vaccine?**  The vaccines approved for use in the UK have met strict standards of safety, quality and effectiveness set out by the independent Medicines and Healthcare products Regulatory Agency (MHRA).  Any COVID-19 vaccine that is approved must go through all the clinical trials and safety checks all other licensed medicines go through. The MHRA follows international standards of safety.  Other vaccines are being developed. They will only be available on the NHS once they have been thoroughly tested to make sure they are safe and effective.  So far, millions of people have been given a COVID-19 vaccine and reports of serious side effects, such as allergic reactions and blood clots, have been very rare. No long-term health complications have been reported.  **How effective is the COVID-19 vaccine?**  The first dose of the COVID-19 vaccine should give you good protection from COVID-19. But you need to have the 2 doses of the vaccine to give you longer-lasting protection.  Researchers think that the vaccines will reduce the number of cases of COVID-19 by between 75% and 94% in those who are vaccinated, based on the results of clinical trials involving 10,000s of volunteers. It is not currently known how long protection lasts. The people vaccinated in clinical trials will continue to be followed for 2 years.  There is a chance you might still get or spread COVID-19 even if you have the vaccine. This means it is important to:   - continue to follow social distancing guidance, - if you can, wear something that covers your nose and mouth in places where it's hard to stay away from other people.   **COVID-19 vaccine side effects**  Most side effects of the COVID-19 vaccine are mild and should not last longer than a week, such as:   - a sore arm where the needle went in, - feeling tired, - a headache, - feeling achy, - feeling or being sick   There are lots of myths about the vaccine: you cannot get COVID-19 from the vaccine, it does not affect your DNA, nor your fertility. The approved COVID-19 vaccines do not contain any animal products or egg. |

### **Supplementary table 3.** Study 1 questionnaire wording

(measures marked with asterisk were also included in Study 2 questionnaire alongside those detailed in Supplementary table 9)

| Measure | Preface | Item(s) | Values | Value Labels |
| --- | --- | --- | --- | --- |
| Prior COVID-19 vaccine beliefs | The currently available COVID-19 vaccines are... | ...safe  ...effective in preventing the disease  ...dangerous [reversed]  ...don't work [reversed] | 1 2 3 4 5 6 7 | Strongly disagree Disagree Somewhat disagree Neither agree nor disagree Somewhat agree Agree Strongly agree |
| Affective reactance* | How did this vaccine information make you feel? For each of the feelings below, please indicate on the sliding scales (from 'Not at all' to 'Extremely'). | Resentful  Bitter  Hateful  Angry | range: 0-100 | |
| Information trustworthiness* | Please tell us to what extent you think the information you read was... | Accurate  Reliable  Trustworthy | 1 2 3 4 5 6 7 | Not at all 2 3 4 5 6 Very much |
| Perceived quality of evidence* |  | How high or low do you think the quality of the evidence underlying the information you just read is? | 1 2 3 4 5 6 7 | Low quality of evidence 2 3 4 5 6 High quality of evidence |
| Engagement* | Still thinking about the information you just read, please answer the questions below. | In your opinion, would other people want to read this?  Are you interested in this information?  Do you like how this information is presented? | 1 2 3 4 5 6 7 | Not at all 2 3 4 5 6 Very much |
| Producer trustworthiness* |  | To what extent do you think the people who are responsible for producing this message are trustworthy? | 1 2 3 4 5 6 7 | Not trustworthy at all 2 3 4 5 6 Very trustworthy |
| Understanding* |  | How completely do you feel you understood the information we just showed you? | 1 2 3 4 5 6 7 | Didn't understand it at all 2 3 4 5 6 Understood it completely |
| Effort* |  | How much effort do you feel you had to put into understanding the information we just showed you? | 1 2 3 4 5 6 7 | None 2 3 4 5 6 A lot |
| Choice threatened | Thinking about the information we showed you, please indicate how much do you agree or disagree with the following statements. | The message threatened my freedom to choose  The message tried to make a decision for me  The message tried to manipulate me | 1 2 3 4 5 6 7 | Strongly disagree Disagree Somewhat disagree Neither agree nor disagree Somewhat agree Agree Strongly agree |
| Cognitive reactance* | Please answer the following questions about the information we showed you. | Did you criticize the message you just saw while you were reading it?  Did you think of points that went against what was being said while you were reading the message?  While reading the message, were you skeptical of what was being said? | 1 2 3 4 5 6 7 | No, not at all 2 3 4 5 6 Yes, very much so |
| Decision confidence (Decisional Conflict Scale; adapted – items in bold in final scale) | If you were given the option of receiving a COVID-19 vaccine or not today how would you feel about the decision? | I know which options are available to me.  I know the benefits of each option.  I know the risks and side effects of each option.  I am clear about which benefits matter most to me.  I am clear about which risks and side effects matter most to me.  I am clear about which is more important to me (the benefits or the risks and side effects).  I have enough support from others to make a choice.  I am choosing without pressure from others.  I have enough advice to make a choice.  I am clear about the best choice for me.  **I feel sure about what to choose.**  **This decision is easy for me to make.**  **I feel I have made an informed choice.**  My decision shows what is important to me.  I expect to stick with my decision.  **I am satisfied with my decision.** | 1 2 3 4 5 | Strongly disagree Somewhat disagree Neither agree nor disagree Somewhat agree Strongly agree |
| Vaccine intentions |  | Would you take a COVID-19 vaccine (approved for use in the UK) if offered? | 1 2 3 4 5 | Definitely Probably I may or I may not Probably not Definitely not |
|  |  | Now that there is a COVID-19 vaccine available in the UK: | 1 2 3 4 5 | I will want to get it as soon as possible I will take it when offered I’m not sure what I will do I will put off (delay) getting it I will refuse to get it |
|  |  | I would describe my attitude towards receiving a COVID-19 vaccine as: | 1 2 3 4 5 | Very keen Pretty positive Neutral Quite uneasy Against it |
|  |  | If a COVID-19 vaccine was available at my local pharmacy, I would: | 1 2 3 4 5 | Get it as soon as possible Get it when I have time Delay getting it Avoid getting it for as long as possible Never get it |
|  |  | If my family or friends were thinking of getting a COVID-19 vaccination, I would: | 1 2 3 4 5 | Strongly encourage them Encourage them Not say anything to them about it Ask them to delay getting the vaccination Suggest that they do not get the vaccination |
|  |  | I would describe myself as: | 1 2 3 4 5 | Eager to get a COVID-19 vaccine Willing to get the COVID-19 vaccine Not bothered about getting the COVID-19 vaccine Unwilling to get the COVID-19 vaccine Anti-vaccination for COVID-19 |
|  |  | Taking a COVID-19 vaccination is: | 1 2 3 4 5 | Really important Important Neither important nor unimportant Unimportant Really unimportant |
| Vaccine acceptance (Binary question) |  | If you were offered a COVID-19 vaccine: - Would you get vaccinated yourself? | 1 2 | Yes No |

### **Supplementary table 4.** Scale reliabilities, means and standard deviations of Study 1 outcomes, across conditions

(including ‘partial’ condition not reported in main text)

| Outcome | α | Persuasive | |  | Partial | |  | Balanced | |
| --- | --- | --- | --- | --- | --- | --- | --- | --- | --- |
|  |  | *M* | *SD* |  | *M* | *SD* |  | *M* | *SD* |
| COVID vaccine prior beliefs | .90 | 5.25 | 1.38 |  | 5.30 | 1.33 |  | 5.30 | 1.31 |
| Information trustworthiness | .96 | 5.28 | 1.50 |  | 5.45 | 1.42 |  | 5.39 | 1.35 |
| Producer trustworthiness |  | 5.16 | 1.53 |  | 5.27 | 1.48 |  | 5.28 | 1.36 |
| Affective reactance | .93 | 17.5 | 24.43 |  | 15.46 | 21.86 |  | 15.47 | 21.51 |
| Cognitive reactance | .80 | 2.83 | 1.70 |  | 2.79 | 1.68 |  | 2.77 | 1.58 |
| Freedom threat | .86 | 3.02 | 1.54 |  | 2.94 | 1.43 |  | 2.95 | 1.43 |
| Engagement | .84 | 5.31 | 1.34 |  | 5.38 | 1.28 |  | 5.49 | 1.22 |
| Perceived quality of evidence |  | 5.02 | 1.6 |  | 5.14 | 1.56 |  | 5.25 | 1.43 |
| Understanding |  | 6.08 | 1.15 |  | 6.12 | 1.11 |  | 5.98 | 1.16 |
| Effort |  | 3.44 | 1.88 |  | 3.42 | 1.82 |  | 3.74 | 1.79 |
| Vaccine intentions | .97 | 4.03 | 1.06 |  | 4.03 | 1.02 |  | 4.06 | 1.01 |
| Decision confidence (full) | .96 | 4.26 | 0.69 |  | 4.23 | 0.74 |  | 4.23 | 0.76 |
| Decision confidence | .89 | 4.28 | 0.79 |  | 4.24 | 0.84 |  | 4.23 | 0.85 |

Cronbach’s α shown for multi-item measures

### **Supplementary table 5.** Study 1 results of one-way ANOVA tests and pairwise comparisons between conditions

(Tukey’s post hoc; includes ‘Partial’ condition not reported in main text).

| Outcome | One-way ANOVA | | | | |  | Pairwise comparisons | | | | |
| --- | --- | --- | --- | --- | --- | --- | --- | --- | --- | --- | --- |
|  | *DFn* | *DFd* | *F* | *p* | η² |  | Group 1 | Group2 | *M*_diff_ | *p* | *d* |
| Information trustworthiness | 2 | 2923 | 3.31 | .04 | 0.002 |  | Persuasive | Partial | **0.16** | **.03** | **0.11** |
|  |  |  |  |  |  |  | Persuasive | Balanced | 0.11 | .21 | 0.08 |
|  |  |  |  |  |  |  | Partial | Balanced | -0.06 | .67 | 0.04 |
| Producer trustworthiness | 2 | 2892 | 2.18 | .11 | 0.002 |  | Persuasive | Partial | 0.11 | .20 | 0.08 |
|  |  |  |  |  |  |  | Persuasive | Balanced | 0.12 | .14 | 0.09 |
|  |  |  |  |  |  |  | Partial | Balanced | 0.01 | .99 | 0.01 |
| Affective reactance | 2 | 2886 | 2.59 | .08 | 0.002 |  | Persuasive | Partial | -2.04 | .12 | 0.09 |
|  |  |  |  |  |  |  | Persuasive | Balanced | -2.02 | .12 | 0.09 |
|  |  |  |  |  |  |  | Partial | Balanced | 0.02 | 1.00 | 0.00 |
| Cognitive reactance | 2 | 2923 | 0.32 | .72 | 0.000 |  | Persuasive | Partial | -0.03 | .91 | 0.02 |
|  |  |  |  |  |  |  | Persuasive | Balanced | -0.06 | .70 | 0.04 |
|  |  |  |  |  |  |  | Partial | Balanced | -0.03 | .92 | 0.02 |
| Freedom threat | 2 | 2923 | 0.85 | 0.43 | 0.001 |  | Persuasive | Partial | -0.08 | .46 | 0.05 |
|  |  |  |  |  |  |  | Persuasive | Balanced | -0.07 | .55 | 0.05 |
|  |  |  |  |  |  |  | Partial | Balanced | 0.01 | .99 | 0.01 |
| Engagement | 2 | 2922 | 4.82 | .01 | 0.003 |  | Persuasive | Partial | 0.07 | .48 | 0.05 |
|  |  |  |  |  |  |  | Persuasive | Balanced | **0.18** | **.01** | **0.14** |
|  |  |  |  |  |  |  | Partial | Balanced | 0.11 | .14 | 0.09 |
| Perceived quality of evidence | 2 | 2918 | 5.29 | .01 | 0.004 |  | Persuasive | Partial | 0.11 | .24 | 0.07 |
|  |  |  |  |  |  |  | Persuasive | Balanced | **0.23** | **< .01** | **0.15** |
|  |  |  |  |  |  |  | Partial | Balanced | 0.11 | .23 | 0.08 |
| Understanding | 2 | 2923 | 4.07 | .02 | 0.003 |  | Persuasive | Partial | 0.04 | .72 | 0.04 |
|  |  |  |  |  |  |  | Persuasive | Balanced | -0.10 | .12 | 0.09 |
|  |  |  |  |  |  |  | Partial | Balanced | **-0.14** | **.02** | **0.13** |
| Effort | 2 | 2924 | 9.23 | < .001 | 0.006 |  | Persuasive | Partial | -0.02 | .97 | 0.01 |
|  |  |  |  |  |  |  | Persuasive | Balanced | **0.30** | **< .001** | **0.16** |
|  |  |  |  |  |  |  | Partial | Balanced | **0.32** | **< .001** | **0.18** |
| Vaccine intentions | 2 | 2920 | 0.24 | .79 | 0.000 |  | Persuasive | Partial | 0.00 | 1.00 | 0.00 |
|  |  |  |  |  |  |  | Persuasive | Balanced | 0.03 | .84 | 0.03 |
|  |  |  |  |  |  |  | Partial | Balanced | 0.03 | .80 | 0.03 |
| Decision confidence | 2 | 2921 | 0.74 | .48 | 0.001 |  | Persuasive | Partial | -0.03 | .65 | 0.04 |
|  |  |  |  |  |  |  | Persuasive | Balanced | -0.04 | .48 | 0.05 |
|  |  |  |  |  |  |  | Partial | Balanced | -0.01 | .96 | 0.01 |
| Decision confidence (full scale) | 2 | 2916 | 0.56 | .57 | 0.000 |  | Persuasive | Partial | -0.03 | .63 | 0.04 |
|  |  |  |  |  |  |  | Persuasive | Balanced | -0.03 | .63 | 0.04 |
|  |  |  |  |  |  |  | Partial | Balanced | 0.00 | 1.00 | 0.00 |

Significant pairwise differences shown in bold

### **Supplementary table 6.** Study 1 full regression results for models examining interaction between message condition and prior beliefs

in predicting outcomes (including ‘partial’ condition not reported in main text).

|  | Info. Trust. | Prod. Trust. | Cog. React. | Aff.  React | Free. Threat | Engage. | Quality | Underst. | Effort | Vacc. Intent. | Dec. conf. [full]^a^ | Dec. conf.^a^ |
| --- | --- | --- | --- | --- | --- | --- | --- | --- | --- | --- | --- | --- |
| Message [Persuasive vs Partial] | 0.08 ^**^ | 0.05 | 0.01 | -0.06 | -0.03 | 0.03 | 0.05 | 0.02 | -0.01 | -0.04 | -0.06 | -0.06 |
|  | [0.02,  0.14] | [-0.01,  0.12] | [-0.06,  0.08] | [-0.14,  0.01] | [-0.10,  0.04] | [-0.04,  0.10] | [-0.02,  0.12] | [-0.06,  0.11] | [-0.10,  0.08] | [-0.09,  0.02] | [-0.14,  0.02] | [-0.14,  0.02] |
| Message [Persuasive vs Balanced] | 0.05 | 0.06 | -0.01 | -0.06 | -0.02 | 0.12 ^**^ | 0.12 ^***^ | -0.10 ^*^ | 0.16 ^***^ | -0.01 | -0.06 | -0.07 |
|  | [-0.01,  0.10] | [-0.01,  0.13] | [-0.08,  0.06] | [-0.14,  0.01] | [-0.10,  0.05] | [0.04,  0.19] | [0.05,  0.19] | [-0.19,  -0.02] | [0.07,  0.25] | [-0.06,  0.05] | [-0.14,  0.02] | [-0.15,  0.01] |
| Prior beliefs | 0.79 ^***^ | 0.73 ^***^ | -0.65 ^***^ | -0.65 ^***^ | -0.61 ^***^ | 0.61 ^***^ | 0.68 ^***^ | 0.29 ^***^ | 0.01 | 0.81 ^***^ | 0.42 ^***^ | 0.38 ^***^ |
|  | [0.75,  0.83] | [0.68,  0.77] | [-0.70,  -0.61] | [-0.70,  -0.60] | [-0.66,  -0.56] | [0.56,  0.66] | [0.64,  0.73] | [0.23,  0.35] | [-0.05,  0.07] | [0.77,  0.84] | [0.36,  0.47] | [0.33,  0.44] |
| Message [Persuasive vs Partial]* Prior beliefs | -0.04 | -0.02 | 0.01 | 0.08 ^*^ | 0.07 | -0.05 | 0.02 | 0.03 | 0.05 | 0.01 | 0.04 | 0.05 |
|  | [-0.10,  0.02] | [-0.08,  0.05] | [-0.06,  0.08] | [0.00,  0.15] | [-0.01,  0.14] | [-0.12,  0.02] | [-0.05,  0.08] | [-0.06,  0.11] | [-0.04,  0.14] | [-0.04,  0.06] | [-0.03,  0.12] | [-0.02,  0.13] |
| Message [Persuasive vs Balanced]* Prior beliefs | -0.13 ^***^ | -0.14 ^***^ | 0.07 ^*^ | 0.14 ^***^ | 0.04 | -0.16 ^***^ | -0.09 ^**^ | 0.03 | -0.06 | -0.02 | 0.11 ^**^ | 0.11 ^**^ |
|  | [-0.19,  -0.07] | [-0.21,  -0.08] | [0.00,  0.14] | [0.07,  0.21] | [-0.03,  0.11] | [-0.23,  -0.08] | [-0.16,  -0.03] | [-0.06,  0.11] | [-0.15,  0.03] | [-0.07,  0.03] | [0.03,  0.18] | [0.03,  0.19] |
| Observations | 2926 | 2895 | 2926 | 2889 | 2926 | 2925 | 2921 | 2926 | 2927 | 2923 | 2919 | 2924 |
| *R*^2^ adjusted | 0.549 | 0.460 | 0.391 | 0.337 | 0.336 | 0.305 | 0.437 | 0.096 | 0.007 | 0.646 | 0.218 | 0.190 |

Standardised regression coefficients shown with 95% CI in square brackets.

Full dependent variable labels (in order): Information trustworthiness, Producer trustworthiness, Cognitive reactance, Affective reactance, Freedom threat, Engagement, Perceived quality of evidence, Understanding, Effort, Vaccine intentions, Decision confidence (full scale), Decision confidence.

^a^Inspection of residuals in these models suggested a U-shaped relationship between prior beliefs and decision confidence. Addition of quadratic term significantly increased variance explained and revealed no significant interactions with condition.

**p* < 0.05, ***p* < 0.01, ****p* < 0.001.

### **Supplementary table 7.** Study 1 group means (including inattentive participants).

| Outcome | Persuasive | | Partial | | Balanced | |
| --- | --- | --- | --- | --- | --- | --- |
|  | *M* | *SD* | *M* | *SD* | *M* | *SD* |
| Information trustworthiness | 5.28 | 1.50 | 5.45 | 1.42 | 5.39 | 1.35 |
| Producer trustworthiness | 5.16 | 1.53 | 5.27 | 1.48 | 5.28 | 1.36 |
| Cognitive reactance | 2.83 | 1.70 | 2.79 | 1.68 | 2.77 | 1.58 |
| Affective reactance | 17.50 | 24.43 | 15.46 | 21.86 | 15.47 | 21.51 |
| Vaccine intentions | 4.03 | 1.06 | 4.03 | 1.02 | 4.06 | 1.01 |

### **Supplementary table 8.** Study 1 main effects (including inattentive participants).

| Outcome | One-way ANOVA | | | | |  | Pairwise comparisons | | | | |
| --- | --- | --- | --- | --- | --- | --- | --- | --- | --- | --- | --- |
|  | *DFn* | *DFd* | *F* | *p* | η² |  | Group 1 | Group2 | *M*_diff_ | *p* | *d* |
| Information trustworthiness | 2 | 3222 | 1.34 | 0.262 | 0.001 |  | Persuasive | Partial | 0.10 | 0.25 | 0.07 |
|  |  |  |  |  |  |  | Persuasive | Balanced | 0.07 | 0.48 | 0.05 |
|  |  |  |  |  |  |  | Partial | Balanced | -0.03 | 0.90 | 0.02 |
| Producer trustworthiness | 2 | 3186 | 1.023 | 0.36 | 0.001 |  | Persuasive | Partial | 0.07 | 0.53 | 0.05 |
|  |  |  |  |  |  |  | Persuasive | Balanced | 0.09 | 0.36 | 0.06 |
|  |  |  |  |  |  |  | Partial | Balanced | 0.02 | 0.96 | 0.01 |
| Cognitive reactance | 2 | 3220 | 0.463 | 0.629 | 0.000 |  | Persuasive | Partial | 0.02 | 0.94 | 0.01 |
|  |  |  |  |  |  |  | Persuasive | Balanced | -0.04 | 0.81 | 0.03 |
|  |  |  |  |  |  |  | Partial | Balanced | -0.07 | 0.61 | 0.04 |
| Affective reactance | 2 | 3170 | 1.788 | 0.167 | 0.001 |  | Persuasive | Partial | -1.70 | 0.22 | 0.07 |
|  |  |  |  |  |  |  | Persuasive | Balanced | -1.63 | 0.25 | 0.07 |
|  |  |  |  |  |  |  | Partial | Balanced | 0.07 | 1.00 | 0.00 |
| Vaccine intentions | 2 | 3218 | 0.224 | 0.799 | 0.000 |  | Persuasive | Partial | -0.02 | 0.89 | 0.02 |
|  |  |  |  |  |  |  | Persuasive | Balanced | 0.01 | 0.98 | 0.01 |
|  |  |  |  |  |  |  | Partial | Balanced | 0.03 | 0.79 | 0.03 |

### **Supplementary table 9.** Study 1 interaction effects (including inattentive participants).

|  | Information trustworthiness | Producer trustworthiness | Cognitive reactance | Affective reactance |
| --- | --- | --- | --- | --- |
| Message (Persuasive vs Balanced) | 0.04 | 0.05 | -0.02 | -0.06 |
|  | [-0.02 – 0.10] | [-0.01 – 0.12] | [-0.08 – 0.05] | [-0.13 – 0.01] |
| Prior beliefs | 0.78 *** | 0.71 *** | -0.66 *** | -0.64 *** |
|  | [0.74 – 0.82] | [0.67 – 0.75] | [-0.71 – -0.61] | [-0.69 – -0.59] |
| Message*Prior beliefs | -0.12 *** | -0.12 *** | 0.07 * | 0.11 ** |
|  | [-0.17 – -0.06] | [-0.19 – -0.06] | [0.00 – 0.14] | [0.04 – 0.18] |
| *N* | 2150 | 2130 | 2150 | 2117 |
| *R*^2^ (adjusted) | 0.534 | 0.427 | 0.392 | 0.344 |

Standardised regression coefficients shown with 95% CI in square brackets.

*p < .05, **p < .01, ***p < .001

### **Supplementary table 10.** Study 2 demographics

| Category | Response | *n* | % |
| --- | --- | --- | --- |
| Gender | Woman | 523 | 50.6 |
|  | Man | 511 | 49.4 |
| Age bracket^a^ | 18-24 | 101 | 9.8 |
|  | 25-34 | 187 | 18.1 |
|  | 35-44 | 179 | 17.3 |
|  | 45-54 | 200 | 19.3 |
|  | 55-64 | 207 | 20.0 |
|  | 65+ | 160 | 15.5 |
| Ethnicity | Asian | 56 | 5.4 |
|  | Black | 21 | 2.0 |
|  | Mixed | 19 | 1.8 |
|  | Other | 8 | 0.8 |
|  | White | 930 | 89.9 |
| Education | No formal education above age 16 | 131 | 12.7 |
|  | Professional or technical qualifications above age 16 | 183 | 17.7 |
|  | School education up to age 18 | 270 | 26.1 |
|  | Degree (Bachelors) or equivalent | 316 | 30.6 |
|  | Degree (Masters) or equivalent | 120 | 11.6 |
|  | Doctorate | 13 | 1.3 |

^a^Participants also reported their age in years (*M* = 47.2, *SD* = 16.1)

### **Supplementary table 11.** Full texts of stimuli in Study 2

| Condition | Text |
| --- | --- |
| Persuasive | The government is in the final stages of deciding whether the UK should build a new nuclear power plant.  The UK has committed to net-zero carbon emissions by 2050. This means replacing – as far as possible - fossil fuels with energy technologies which do not release carbon into the atmosphere.  Nuclear power, which is a reliable and low-carbon source of power, has a key role to play, alongside other technologies such as renewables as the UK moves towards net-zero greenhouse gas emissions over the next 30 years.  It is a technology which provides large volumes of power from very little land area at low levels of carbon emissions. A key benefit of nuclear power is that it can provide a consistent level of energy in response to consumer demand, whereas renewable sources of energy like solar and wind power are dependent on weather conditions. The UK needs a source of power that can ensure we can keep the lights on whatever the weather.  A large-scale nuclear power plant will also support a peak of around 10,000 jobs during construction, as well as providing ongoing local jobs at a range of different skill levels. The nuclear industry has set a goal to reduce the cost of nuclear new build projects by 30 per cent by 2030. This cost reduction would be passed on in the cost of the electricity produced by nuclear power.   The UK is therefore keen to weigh up the potential for large-scale new nuclear projects, subject to value-for-money, supported by development funding from the government.  Building a new, state-of-the-art nuclear power station in the UK would be an ambitious project, but could bring investment into the country, and provide jobs and wealth as well as helping to reduce carbon emissions and so help reduce climate change.  As a reliable, low-carbon source of energy, nuclear power therefore has an important role to play in helping the UK achieve net zero by 2050. |
| Balanced | The government is in the final stages of deciding whether the UK should build a new nuclear power plant.  Nuclear power has benefits and drawbacks. The UK has committed to net-zero carbon emissions by 2050. This means replacing - as far as possible - fossil fuels with energy technologies which do not release carbon into the atmosphere.  Nuclear power is an energy-dense technology which means it provides large volumes of power from very little land area at low levels of carbon emissions. Nuclear power plants do not produce carbon emissions during operation: the heat produced by nuclear materials is used to boil water, creating steam to drive turbines.  Another key benefit of nuclear power is that it can provide a consistent level of energy, whereas sources like solar and wind power are dependent on weather.  Building a new nuclear power plant could support jobs during construction. Estimates based on previous nuclear power plant builds suggest this could peak at around 10,000 jobs, but the evidence behind these estimates is of low quality.  A key drawback of nuclear power is that it is more expensive than renewable sources. Government modelling suggests that in 2025 nuclear power will cost between £91 and £132 per megawatt hour compared to between £39 and £52 for onshore wind power and between £39 and £51 for solar power.  The nuclear industry has set a goal to reduce the cost of nuclear new build projects by 30 per cent by 2030 to make the costs more competitive. However, one government report estimates that the future costs of renewable energy are likely to drop faster than the costs of nuclear power.  An important challenge of nuclear power is the need to operate and to store radioactive waste safely. Currently most radioactive nuclear waste is stored in a purpose-built facility in the UK, with plans to build a larger underground facility in the future. |

### **Supplementary table 12.** Study 2 additional questionnaire items wording

(see supplementary table 3 for other measures that were unchanged)

| Measure | Preface | Item[s) | Values | Value Labels |
| --- | --- | --- | --- | --- |
| Prior nuclear beliefs |  | From what you know nuclear power for generating electricity in the UK, on balance, which of these statements most closely reflects your own opinion? | 1  2  3  4  5 | The benefits of nuclear power far outweigh the risks The benefits of nuclear power slightly outweigh the risks The benefits and risks of nuclear power are about the same The risks of nuclear power slightly outweigh the benefits The risks of nuclear power far outweigh the benefits |
|  |  | On a purely emotional level, how do you personally feel about nuclear power? | 1 2 3 4 5 | Very positive Fairly positive Neither positive nor negative Fairly negative Very negative |
|  |  | How favourable or unfavourable is your overall opinion or impression of nuclear power for producing electricity currently? | 1 4 5 6 7 | Very favourable Mainly favourable Neither favourable nor unfavourable Mainly unfavourable Very unfavourable |
| Ratings of meeting Blastland et al. criteria | For each of the questions below, please move the slider to a location on the line between the two points to indicate your answer. | Do you think the message aimed to persuade or inform you? | 0  100 | Persuade  Inform |
|  |  | Was the information one-sided or balanced? | 0  100 | One-sided  Balanced |
|  |  | Did the message acknowledge the quality of the evidence behind claims? | 0  100 | Did not acknowledge quality  Did acknowledge quality |
|  |  | Did the message present information as relatively more certain or uncertain? | 0  100 | Certain  Uncertain |
|  |  | Did the message try to address possible misunderstandings about the topic? | 0  100 | Did not address misunderstandings  Did address misunderstandings |
|  |  | The message presented a range of potential scenarios when talking about benefits and costs. | 0  100 | Completely disagree  Completely agree |
| Nuclear policy support |  | How much do you support or oppose plans to build a new nuclear power plant in the UK? | 1 2 3 4 5 6 7 | Strongly oppose Oppose Somewhat oppose Neither oppose nor support Somewhat support Support Strongly support |
| Decision confidence (Decisional Conflict Scale) | We just asked you how much you support or oppose building a new nuclear power plant in the UK. How do you feel about your decision to support or oppose such plans? | I felt sure about what to choose.  This decision was easy for me to make.  I feel I have made an informed decision.  I am satisfied with my decision. | 1 2 3 4 5 | Strongly disagree Somewhat disagree Neither agree nor disagree Somewhat agree Strongly agree |
| Subjective knowledge |  | How well do you feel you know the potential risks and benefits of nuclear power? | 1 2 3 4 5 6 7 | Not at all 2 3 4 5 6 Very much |

### **Supplementary table 13.** Scale reliabilities, means and standard deviations of Study 2 outcomes, across conditions, and t-test results.

| Outcome | α | Persuasive | | |  | Balanced | | | *p* | *d* |
| --- | --- | --- | --- | --- | --- | --- | --- | --- | --- | --- |
|  |  | *M* | *SD* | |  | *M* | *SD* | |  |  |
| Nuclear prior beliefs | 0.92 | 3.33 | | 1.07 |  | 3.20 | | 1.07 | .05 | 0.12 |
| Information trustworthiness | 0.92 | 4.70 | | 1.33 |  | 4.92 | | 1.19 | < .01 | 0.17 |
| Producer trustworthiness |  | 4.35 | | 1.47 |  | 4.63 | | 1.31 | < .01 | 0.20 |
| Affective reactance | 0.92 | 25.79 | | 24.08 |  | 28.21 | | 24.68 | .11 | 0.10 |
| Cognitive reactance | 0.83 | 3.48 | | 1.53 |  | 3.41 | | 1.45 | .41 | 0.05 |
| Engagement | 0.82 | 4.96 | | 1.30 |  | 5.06 | | 1.24 | .20 | 0.08 |
| Understanding |  | 5.41 | | 1.26 |  | 5.47 | | 1.21 | .38 | 0.05 |
| Effort |  | 3.82 | | 1.66 |  | 3.92 | | 1.65 | .34 | 0.06 |
| Perceived quality of evidence |  | 4.53 | | 1.44 |  | 4.63 | | 1.40 | .30 | 0.07 |
| Nuclear policy support |  | 4.56 | | 1.71 |  | 4.16 | | 1.73 | < .001 | 0.23 |
| Decision confidence | 0.87 | 3.96 | | 0.82 |  | 3.95 | | 0.81 | .73 | 0.02 |
| Subjective knowledge |  | 4.69 | | 1.42 |  | 4.77 | | 1.43 | .40 | 0.05 |

Cronbach’s α shown for multi-item measures, Cohen’s d reported as effect size.

### **Supplementary table 14.** Study 2 cue detection slider item details, condition means and SD, and t-test results.

| Item text | Left anchor (0) | Right anchor (100) | Persuasive | |  | Balanced | | *p* | *d* |
| --- | --- | --- | --- | --- | --- | --- | --- | --- | --- |
|  |  |  | *M* | *SD* |  | *M* | *SD* |  |  |
| Do you think the message aimed to **persuade** or **inform** you? | Persuade | Inform | 53.51 | 31.47 |  | 68.66 | 26.58 | < .001 | 0.52 |
| Was the information **one-sided** or **balanced**? | One-sided | Balanced | 52.21 | 29.54 |  | 66.78 | 26.00 | < .001 | 0.52 |
| Did the message acknowledge the **quality of the evidence** behind claims? | Did not acknowledge quality | Did acknowledge quality | 56.42 | 25.95 |  | 62.93 | 25.00 | < .001 | 0.26 |
| Did the message present information as relatively more **certain** or **uncertain**? | Certain | Uncertain | 33.33 | 23.59 |  | 39.12 | 23.69 | < .001 | 0.24 |
| Did the message try to **address possible misunderstandings** about the topic? | Did not address misunderstandings | Did address misunderstandings | 52.44 | 28.02 |  | 60.68 | 24.36 | < .001 | 0.31 |
| The message presented **a range of potential scenarios** when talking about benefits and costs. | Completely disagree | Completely agree | 58.40 | 25.49 |  | 68.34 | 20.90 | < .001 | 0.43 |

Cohen’s *d* reported as effect size.

### **Supplementary table 15.** Study 2 full regression results for models examining interaction between message condition and prior beliefs in predicting outcomes.

|  | Info. trust. | Prod. trust. | Cog. react. | Affect. React | Engage. | Underst. | Effort | Quality | Policy supp. | Dec. conf.^a^ | Subj. know. |
| --- | --- | --- | --- | --- | --- | --- | --- | --- | --- | --- | --- |
| Message (Persuasive vs Balanced) | 0.22 ^***^ | 0.25 ^***^ | -0.1 | 0.07 | 0.12 ^*^ | 0.07 | 0.06 | 0.11 | -0.14 ^***^ | 0 | 0.07 |
|  | [0.11,  0.33] | [0.14,  0.36] | [-0.21,  0.01] | [-0.05,  0.19] | [0.01,  0.24] | [-0.05,  0.19] | [-0.06,  0.18] | [-0.00,  0.22] | [-0.21,  -0.06] | [-0.12,  0.12] | [-0.05,  0.20] |
| Prior beliefs | 0.56 ^***^ | 0.52 ^***^ | -0.48 ^***^ | -0.26 ^***^ | 0.41 ^***^ | 0.16 ^***^ | -0.03 | 0.46 ^***^ | 0.76 ^***^ | 0.29 ^***^ | 0.22 ^***^ |
|  | [0.48,  0.63] | [0.44,  0.59] | [-0.56,  -0.40] | [-0.34,  -0.17] | [0.33,  0.49] | [0.08,  0.25] | [-0.12,  0.06] | [0.38,  0.54] | [0.71,  0.82] | [0.21,  0.38] | [0.13,  0.30] |
| Message (Persuasive vs Balanced)*Prior beliefs | -0.24 ^***^ | -0.20 ^***^ | 0.17 ^**^ | -0.01 | -0.14 ^*^ | -0.01 | 0.03 | -0.16 ^**^ | 0.02 | -0.19 ^**^ | -0.07 |
|  | [-0.35,  -0.13] | [-0.31,  -0.09] | [0.06,  0.28] | [-0.13,  0.11] | [-0.25,  -0.02] | [-0.13,  0.11] | [-0.09,  0.16] | [-0.28,  -0.05] | [-0.05,  0.10] | [-0.30,  -0.07] | [-0.19,  0.05] |
| N | 1030 | 1034 | 1033 | 1034 | 1028 | 1034 | 1033 | 1034 | 1034 | 1034 | 1034 |
| *R*^2^ adjusted | 0.209 | 0.190 | 0.162 | 0.068 | 0.118 | 0.023 | -0.002 | 0.145 | 0.607 | 0.046 | 0.032 |

Standardised regression coefficients shown with 95% CI in square brackets.

Full dependent variable labels (in order): Information trustworthiness, Producer trustworthiness, Affective reactance, Cognitive reactance, Engagement, Understanding, Effort, Perceived quality of evidence, Support for new nuclear power plant, Decision confidence, Subjective knowledge.

^a^Inspection of residuals in this model suggested a U-shaped relationship between prior beliefs and decision confidence. Addition of a quadratic term significantly increased variance explained and revealed no significant interactions with message condition.

**p* < 0.05, ***p* < 0.01, ****p* < 0.001

### **Supplementary table 16.** Study 2 group means and main effects (including inattentive participants)

| Outcome | Persuasive | | | Balanced | | | *p* | *d* |
| --- | --- | --- | --- | --- | --- | --- | --- | --- |
|  | *M* | | *SD* | *M* | | *SD* |  |  |
| Information trustworthiness | 4.66 | 1.34 | | 4.81 | 1.25 | | 0.04 | 0.12 |
| Producer trustworthiness | 4.38 | 1.48 | | 4.60 | 1.36 | | < .01 | 0.16 |
| Affective reactance | 29.82 | 25.16 | | 31.31 | 24.96 | | 0.28 | 0.06 |
| Cognitive reactance | 3.62 | 1.53 | | 3.49 | 1.45 | | 0.12 | 0.09 |

### **Supplementary table 17.** Study 2 interaction effects (including inattentive participants)

|  | Information trustworthiness | Producer trustworthiness | Cognitive reactance | Affective reactance |
| --- | --- | --- | --- | --- |
| Message (Persuasive vs Balanced) | 0.15 ** | 0.19 *** | -0.11 * | 0.04 |
|  | [0.05 – 0.25] | [0.09 – 0.29] | [-0.22 – -0.01] | [-0.06 – 0.15] |
| Prior beliefs | 0.51 *** | 0.48 *** | -0.35 *** | -0.21 *** |
|  | [0.44 – 0.58] | [0.41 – 0.55] | [-0.43 – -0.28] | [-0.28 – -0.13] |
| Message (Persuasive vs Balanced)*Prior beliefs | -0.22 *** | -0.16 ** | 0.12 * | 0.02 |
|  | [-0.32 – -0.12] | [-0.26 – -0.06] | [0.02 – 0.23] | [-0.09 – 0.12] |
| *N* | 1297 | 1305 | 1304 | 1305 |
| *R*^2^ adjusted | 0.174 | 0.167 | 0.088 | 0.038 |

Standardised regression coefficients shown with 95% CI in square brackets.

**p* < 0.05, ***p* < 0.01, ****p* < 0.001

# Supplementary figures


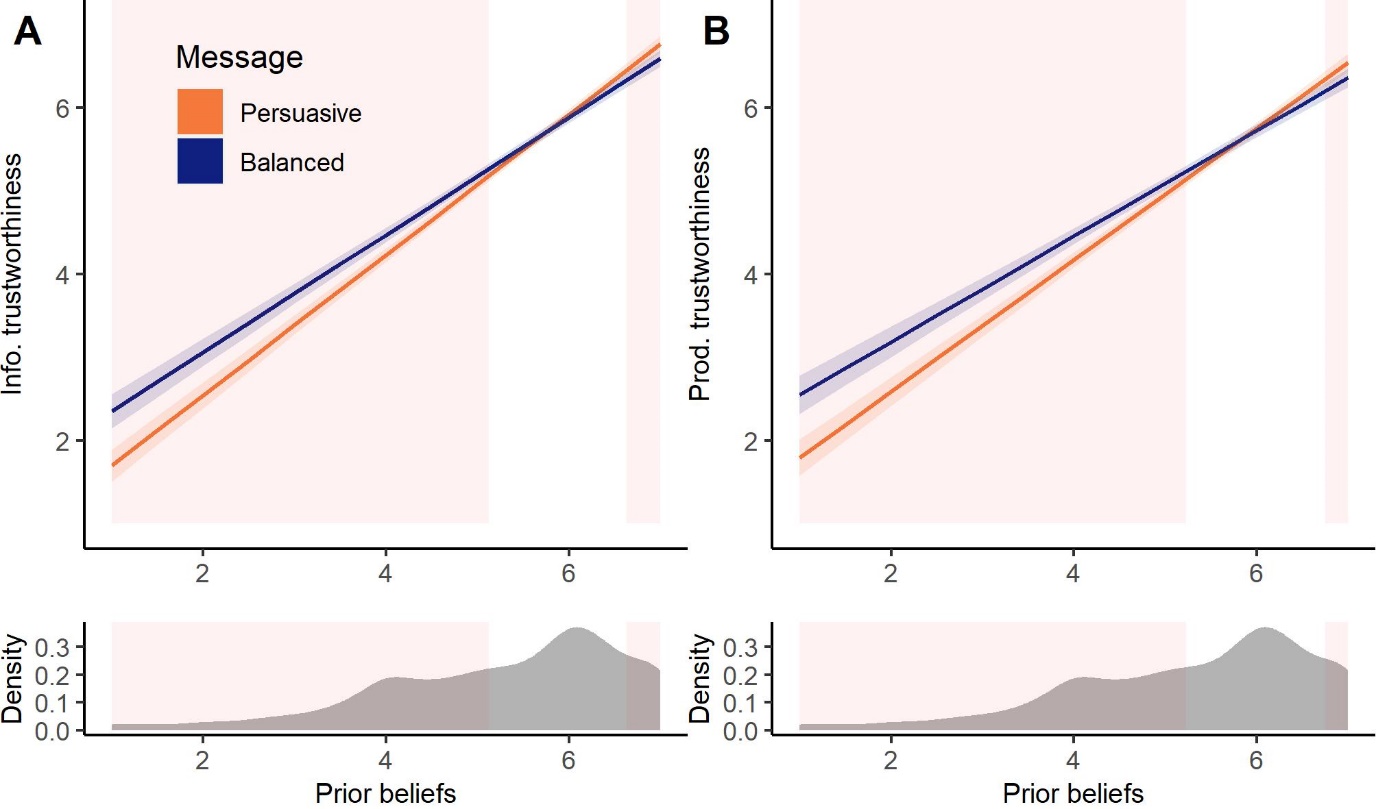


### **Supplementary Figure 1.** Study 1 interaction plots with distribution of prior belief scores.

Upper plots show predicted values (with 95% CI) of information trustworthiness (A) and producer trustworthiness (B) in each condition across the spectrum of prior beliefs for study topic (higher values indicate more positive beliefs). Shaded regions indicate values of prior belief at which the effect of message condition is significant (*p* < .05), based on Johnson-Neyman intervals. Lower plots show the distribution of scores on the prior beliefs scale, with this interval superimposed, providing an indication of the proportion of the sample falling above or below the interval limits.
